# Extracellular RNAs in Liquid Biopsy: Applications in MASLD and MASH Diagnosis and Monitoring

**DOI:** 10.3390/diagnostics15182346

**Published:** 2025-09-16

**Authors:** Dimitrios Raptis, Shiny Teja Kolli, Sonal Agarwal, Praveen Kumar Komminni, Prashamsha Bhattarai, Prinka Hablani, Rahul Kumar, Petras Das, Martin McIntosh, Michail Kladas, Priyanka Gokulnath, Michail Spanos

**Affiliations:** 1 NYC Health and Hospitals/Jacobi/North Central Bronx, Bronx, NY 10461, USA; raptisd1@nychhc.org (D.R.); shinykolli.0393@gmail.com (S.T.K.); sagarwal.us@gmail.com (S.A.); komminnipraveen@gmail.com (P.K.K.); dr.prashamsha@gmail.com (P.B.); hablaniprinka8@gmail.com (P.H.); rbatra390@gmail.com (R.K.); drpetraslohana@gmail.com (P.D.); martin.n.mcintosh@gmail.com (M.M.); kladasm@nychhc.org (M.K.); 2 Cardiovascular Research Center, Massachusetts General Hospital and Harvard Medical School, Boston, MA 02114, USA

**Keywords:** liquid biopsy, extracellular RNAs, microRNAs, extracellular vesicles, metabolic dysfunction-associated steatotic liver disease, metabolic dysfunction-associated steatohepatitis, liver fibrosis

## Abstract

Metabolic dysfunction-associated steatotic liver disease (MASLD) is an increasingly prevalent condition linked to obesity, diabetes, and metabolic syndrome, and can progress to fibrosis, cirrhosis, and hepatocellular carcinoma. Current diagnostic standards such as liver biopsy are invasive and unsuitable for routine screening. Liquid biopsy, particularly through analysis of extracellular RNAs (exRNAs), including microRNAs (e.g., miR-122, miR-21, miR-34a), long non-coding RNAs, and tRNA-derived fragments, offers a promising non-invasive alternative. These exRNAs, released from hepatocytes and carried in blood via extracellular vesicles or protein complexes, can be detected using techniques like RNA sequencing, qRT-PCR, and droplet digital PCR. These biomarkers correlate with histologic severity, fibrosis stage, and treatment response, and have shown promising diagnostic utility; however, their performance may differ across various populations and disease stages. Despite their potential, clinical translation is limited by a lack of standardization and large-scale validation. This review outlines recent advances in exRNA-based diagnostics for MASLD and MASH, emphasizing their role in early detection, disease monitoring, and the shift toward personalized hepatology.

## 1. Introduction

Metabolic dysfunction-associated steatotic liver disease (MASLD) has emerged as the most prevalent chronic liver condition globally, affecting 38% of adults and 7–14% of children, with projections suggesting its prevalence will exceed 55% among adults by 2040 [1,2]. This alarming growth parallels rising rates of diabetes, obesity, and metabolic syndrome, positioning MASLD to become the leading cause of liver transplantation in the U.S. within the next decade [3,4,5]. The economic burden is equally staggering: A 2024 Italian study estimated annual healthcare costs attributable to MASLD complications at €12.25 billion, with 13,438 excess deaths annually compared to non-MASLD populations [6]. In 2023, a multinational consensus led by hepatology societies redefined the condition from nonalcoholic fatty liver disease (NAFLD) to MASLD, initially proposed in 2020 [7], emphasizing its metabolic etiology through revised diagnostic criteria requiring at least one cardiometabolic risk factor [8]. MASLD is defined as the former NAFLD with at least one cardiometabolic factor and no other secondary cause of steatosis. This nomenclature shift aims to reduce the stigma while highlighting the disease’s systemic nature, since 48.3% of global liver cirrhosis cases now originate from MASLD, with Asia bearing the highest burden [2]. Beyond hepatic outcomes, MASLD independently increases risks for cardiovascular events, chronic kidney disease, and extrahepatic cancers, necessitating multidisciplinary management strategies [9].

Current diagnostic reliance on liver biopsy faces challenges due to invasiveness and sampling variability. While magnetic resonance imaging (MRI) and ultrasound elastography provide noninvasive alternatives, their moderate accuracy for early-stage fibrosis [area under the curve (AUC) 0.70–0.86] limits utility in population screening [10,11]. It is broadly accepted that MASLD diagnosis and treatment in the future will be based on personalized molecular signature [12,13].

Liquid biopsies, which involve the measurement of disease-associated cell-free DNA (cfDNA), long non-coding RNA (lncRNA), extracellular RNA (exRNA), proteins and circulating extracellular vesicles (EVs) from body fluids, have currently emerged as promising diagnostic tools, valuable for monitoring MASLD progression. cfDNAs, fragmented DNA machinery released into the bloodstream by apoptotic and necrotic cells, are associated with MASLD severity [14,15], and further even specific histone signatures appear to play a role in lean MASLD [16]. However, due to their high degree of fragmentation, their utilization as biomarkers can be problematic [17], exhibiting low specificity for MASH [18]. For these reasons, their investigation will be out of the scope of this study. On the contrary, liquid biopsy platforms analyzing exRNA profiles in biofluids offer transformative potential, as demonstrated by miR-122, a liver-specific microRNA showing 2.4-fold higher sensitivity than ALT for detecting drug-induced injury and correlating with histopathological severity scores [19,20]. Notably, certain microRNA (miRNA) subtypes, in combination with leptin, a hormone that is related to MASLD [21], have shown remarkable accuracy in evaluating steatosis, liver stiffness, and hepatic fat content [22]. Recent advancements in EV isolation enable detection of steatosis-associated miRNAs like miR-192-5p and miR-34a, both capable of distinguishing MASH from simple steatosis in multi-center trials [3,23,24]. ExRNAs hold promise not only as diagnostic and monitoring tools, but also as potential novel therapeutic targets to combat the growing epidemic of MASLD [25,26]. Their dysregulation has been linked to diseases such as inflammatory bowel disease (IBD), colorectal cancer, and MASLD. For example, in IBDs, miRNAs modulate inflammatory signaling, epithelial barrier integrity, immune responses, and autophagy [27], while at the same time they act as regulators of tumor apoptosis, proliferation and progression in the setting of colorectal cancer. This review synthesizes evolving evidence on exRNA biomarkers in MASLD diagnostics, focusing on their role in staging fibrosis, predicting treatment response, and enabling precision hepatology through minimally invasive serial monitoring.

## 2. Extracellular RNA as Carrier of Circulating Biomarkers

### 2.1. Types of Extracellular RNAs

ExRNAs represent a diverse molecular ecosystem within biofluids, with distinct subtypes demonstrating unique roles in MASLD pathogenesis. ExRNAs can be found in almost all biological fluids including venous blood, cerebrospinal fluid, breast milk, pleural fluid, urine and saliva. Similar to the different RNA species, there are different types of exRNAs, including coding or messenger RNA (mRNA), and various non-coding RNAs such as miRNA, lncRNA, transfer RNA (tRNA) and circular RNA (circRNA) among others (Table 1). These exRNAs can be present either as full-length molecules or as fragments in the circulating biofluids with relevant RNA-base modifications. One of the highly prevalent classes of exRNAs is mRNAs which are responsible for protein synthesis.

MiRNAs, one of the most common and well-studied types of circulating exRNAs, are small (19–25 nucleotides), non-coding RNAs that modulate gene expression post-transcriptionally [28]. miRNAs are among the most abundant RNA species in plasma and serum with the exception of ribosomal RNAs [49]. miRNAs can be packaged into EVs and transferred between cells, allowing direct intercellular communication. Several miRNAs, such as miR-122, miR-34a, miR-21, miR-145, miR-451, miR-27b-3p, miR-30a-5p, miR-375-3p, miR-103a-3p, let-7d-5p, let-7f-5p, have been linked to hepatic steatosis and MASH [29].

lncRNAs refer to a class of RNA molecules that are longer than 200 nucleotides and do not encode proteins, comprising the majority of the non-coding RNA. lncRNAs regulate gene expression through various mechanisms, including chromatic modification, transcriptional regulation, and post-transcriptional processing [35,36]. LncRNAs are also being explored for their biomarker potential in various diseases, particularly their circulating counterparts, which can serve as liquid biopsy markers for different conditions [50].

tRNAs, on the other hand, play a critical role in cellular function, since they help translate the sequence of mRNA into corresponding amino acid sequences during protein synthesis, effectively facilitating the construction of proteins based on genetic instructions [44]. Recently, a new type of tRNA has emerged, called tRNA-derived small RNAs or tDRs, which are produced after cleavage at specific sites in the codon or anti-codon loop by restrictive enzymes such as angiogenin (ANG), responsible for cleaving the anticodon loop of mature tRNAs under stressful conditions (such as hypoxia, lack of amino acids, oxidative stress, ultraviolet radiation, heat shock, and viral infection) [51,52].

Small interfering RNAs (SiRNAs) can mediate RNA interference by binding to complementary mRNAs, leading to their degradation and affecting protein synthesis [42].

Lastly, circRNA is generated by back-splicing events and have a covalently closed structure and exhibit high stability in circulation [46]. Their role in MASLD and MASH is still under active investigation, but preliminary findings suggest they can act as microRNA sponges or modulators of transcription and translation [53].

### 2.2. Sources of exRNA in Liquid Biopsies

Liquid biopsies represent a minimally invasive alternative to traditional tissue biopsies, enabling the analysis of biomarkers, including exRNAs and circulating tumor cells in bodily fluids [54]. RNA is a fragile molecule that needs to be safeguarded from the disrupting and degrading action of circulating enzymes found in the extracellular environment and biofluids. The molecules that encapsulate and protect RNA are called RNA carriers. ExRNAs in liquid biopsies originate from various carriers, each offering unique insights into physiological and pathological conditions. One type of RNA carriers is EV which include exosomes, microvesicles, and apoptotic bodies. These membrane-bound particles, released by cells into bodily fluids, encapsulate diverse RNA species such as mRNAs and miRNAs, thereby protecting them from ribonucleases [55] and provide a stable source of genetic material for analysis. Notably, EVs have been identified as carriers of cancer-derived RNAs, presenting potential biomarkers for early cancer detection and monitoring [56]. EVs encapsulate diverse RNA cargo (miRNAs, mRNAs, lncRNAs, circRNAs, etc.) reflective of their cell of origin. Recent research has solidified EVs as rich sources of disease biomarkers. For example, a 2024 study isolated EVs from human serum and found specific microRNAs in those EVs that mirror atherosclerotic plaque composition, suggesting that EV miRNA profiles can indicate plaque development [57]. Methodologically, EV isolation often involves ultracentrifugation, size-exclusion chromatography, or precipitation kits, followed by RNA sequencing or Polymerase Chain Reaction (PCR) to profile the cargo.

Not all circulating exRNA is vesicle-bound. A substantial fraction is associated with soluble RNP complexes, where RNA is bound to RNA-binding proteins (RBPs). Classic examples are Argonaute2 (Ago2) proteins carrying microRNAs in the bloodstream [58]. These Ago2–miRNA complexes are highly abundant in plasma and very stable. Until recently, only a few exRNA–RNP carriers were well characterized (e.g., Ago2, nucleophosmin 1) [59]. New high-throughput methods are now mapping these carriers: for instance, crosslinking immunoprecipitation and sequencing was applied to chart exRNA–protein interactions across biofluids and 128 distinct extracellular RBPs and their RNA cargo were identified, laying the foundation for a new class of liquid biopsy biomarkers [60]. Importantly, an exRNA in an RNP complex offers a dual marker—the RNA and its binding protein. Detecting both can improve specificity for disease diagnosis. As a result, soluble exRNP complexes (like Ago2-miRNA or other RBP-RNA complexes) are gaining attention for their potential to indicate disease states (e.g., inflammation, cancer) when specific RNA-protein pairs are dysregulated [60].

Circulating lipoproteins, especially high-density lipoproteins (HDL), are another overlooked carrier of exRNA. HDL particles can bind small RNAs (primarily miRNAs) and protect them from RNases in blood [61]. Notably, HDL-bound miRNAs are functional: early studies showed that HDL can deliver miRNAs to recipient cells, altering gene expression [61]. For example, HDL transfers miR-223 to endothelial cells to suppress intercellular adhesion molecule-1 (ICAM-1), a cell adhesion molecule, demonstrating a mechanism of intercellular communication via lipoproteins [62]. In conditions like familial hypercholesterolemia, distinct miRNA profiles have been observed in patient HDL particles, highlighting that disease states can alter lipoprotein-associated RNAs [63].

Beyond the well-known carriers above, emerging sources of exRNA have been identified in recent years. Exomeres, first described around 2018, are nanoscale (~30–50 nm) non-membranous particles that are distinct from classical EVs. They lack a lipid bilayer and consist mainly of proteins and nucleic acids. Exomeres were first isolated using advanced fractionation (asymmetric flow field-flow fractionation) to separate them from larger vesicles [64]. They are now recognized as part of the diverse exRNA carrier spectrum, though their specific biological functions are still under investigation [65]. A few years later, in 2021, researchers identified supermeres, an even smaller and denser class of extracellular nanoparticles [66]. Supermeres are isolated by ultracentrifuging the soluble fraction left after removing larger EVs and exomeres [66]. Emerging evidence showed that supermeres are replete with disease-related cargo, including proteins and RNAs tied to cancer chemoresistance [67]. This finding suggests that important biomarker molecules might concentrate in supermeres.

Surprisingly, entire organelles can be released into circulation. A landmark 2020 study demonstrated that human blood contains intact cell-free mitochondria, complete with mitochondrial RNA and DNA [68,69]. These extracellular mitochondria were shown to be respiratory-competent and enclosed by mitochondrial membranes [68], although conflicting data exists regarding whether these mitochondria are fully functional [68]. Nonetheless, the discovery of extracellular mitochondria expands the concept of exRNA carriers, since mitochondria contain their own RNAs and raises questions about their role in signaling. Extracellular mitochondria have been identified in different forms and may serve as unconventional biomarkers for various diseases, including cancer, cardiometabolic disorders, and neurological conditions [70,71,72,73].

By analyzing these diverse sources of exRNA, liquid biopsies offer a non-invasive and dynamic approach for monitoring disease progression, detecting early biomarkers, and assessing treatment responses in real-time. The ability to assess exRNA profiles from various carriers such as extracellular vesicles, ribonucleoprotein complexes, lipoprotein particles, and even extracellular organelles, enhances the sensitivity and specificity of liquid biopsy-based diagnostics. These advantages position liquid biopsies as a powerful tool in precision medicine, enabling early detection, personalized treatment strategies, and improved disease management.

### 2.3. exRNA Detection and Analysis Methods

High-throughput RNA sequencing (RNA-seq) has become a powerful tool to profile the diverse landscape of exRNAs in liquid biopsy samples [74]. Because exRNAs represent fragmented transcripts from various cell types, sequencing approaches have been adapted to capture even low-abundance RNA species. Recent advances enable whole-transcriptome analysis of exRNA from small input volumes that were previously challenging (Table 2). For instance, Small Input Liquid Volume Extracellular RNA Sequencing (SILVER-seq) was introduced to sequence exRNA from a single drop of human serum [75]. Other specialized library prep methods like PALM-Seq (Polyadenylation Ligation Mediated Sequencing) extend RNA-seq to non-polyadenylated exRNAs. PALM-Seq can capture miRNAs, tRNAs, and other RNAs by adding a poly(A) tail, enabling sequencing of a broader exRNA repertoire [76]. These approaches have improved the detection of both small RNAs and mRNAs circulating in biofluids. A comprehensive 2024 review notes that next-generation sequencing (NGS) of exRNA has been applied to monitor cancer progression and even predict pregnancy-related conditions [77]. The sensitivity of NGS allows discovery of novel RNA biomarkers and mutations in cell-free mRNAs that might be missed by targeted assays [77]. Additionally, improved bioinformatics pipelines (e.g., the exceRpt pipeline and exRNA Atlas data standards) address the unique challenges of exRNA data, such as removing contaminants and accounting for the fragmented nature of plasma RNA [78,79]. Overall, advancements in exRNA sequencing over the last five years have expanded our view of the “cell-free transcriptome,” enabling unbiased detection of disease signals. By capturing dozens to hundreds of RNA markers simultaneously, RNA-seq boosts liquid biopsy sensitivity and provides insights (like tissue-of-origin of the RNA) that enhance diagnostic accuracy. This method has been streamlined in other studies to identify new exRNA targets from patient plasma that could have biomarker potential [80,81]. While exRNA-seq profiles molecules outside cells, single-cell RNA sequencing (scRNA-seq) profiles the transcripts within individual cells. In liquid biopsy contexts, scRNA-seq has been applied to rare circulating tumor cells and immune cells, complementing exRNA analysis. For example, scRNA-seq of circulating tumor cells can reveal gene expression signatures of metastatic cells, while exRNA in plasma reflects signals from tumor and stromal cells that may not be captured by analyzing limited cells [82]. Recent studies integrate single-cell or single-nucleus RNA-seq with exRNA-seq to map intercellular communication: one of those combined tumor single-nucleus RNA profiles with exosome small RNA profiles to identify which cell populations likely secreted specific miRNAs, constructing a cell-exRNA network for pancreatic cancer (demonstrating a novel way to infer biomarker sources) [83]. Recent EV cargo analysis methods are pushing sequencing to the single-vesicle level. In 2022, researchers reported the first high-throughput transcriptomic analysis of individual EVs, highlighting heterogeneity in exRNA cargo between vesicles [84].

Such “single-EV sequencing” is still nascent, but early results underscore that even EVs from the same sample carry distinct RNA molecules. As microfluidic sorting and barcoding methods improve, profiling RNAs from single exosomes or microvesicles could become feasible. In summary, scRNA-seq and related low-input sequencing innovations of the past few years provide a deeper context for exRNA findings, helping to link extracellular signals to specific cells and thereby refining the sensitivity and specificity of liquid biopsy interpretations.

Quantitative reverse-transcription PCR (qRT-PCR) has long been a gold standard for detecting specific RNAs due to its sensitivity, specificity, affordability and speed. It remains widely used to validate exRNA biomarkers (e.g., measuring a candidate mRNA or miRNA in plasma) that were discovered by RNA sequencing and analysis. However, conventional qRT-PCR can struggle with the ultra-low RNA concentrations typical of liquid biopsies. In the last five years, the adoption of droplet digital PCR (ddPCR) has markedly improved detection limits for exRNAs. Droplet digital PCR partitions a sample into thousands of nanoliter droplets, each ideally containing zero or one target molecule, followed by end-point PCR in each droplet [85]. This effectively dilutes out background noise and allows absolute quantification of target copies by counting positive droplets. ddPCR greatly increases sensitivity for low-copy exRNAs: for instance, an exosomal miRNA (miR-29) in urine could be detected at <50 copies/µL by ddPCR, whereas standard qPCR required ~6500 copies/µL for detection [86]. Such improvements (over 100-fold sensitivity gain in that case) enable reliable measurement of rare transcripts. ddPCR also shows better precision and reproducibility for serum miRNA quantification compared to qRT-PCR [87]. Researchers have applied RT-ddPCR to measure clinically relevant exRNAs like androgen receptor splice variant 7 (AR-V7) mRNA in exosomes of prostate cancer patients, detecting this therapy-resistance marker in plasma with high sensitivity [88]. The ability to absolutely quantitate exRNA levels without a standard curve is another advantage of ddPCR, improving comparability between labs [89]. In sum, integrating digital PCR into exRNA workflows over the last few years has enhanced reliability (through precise quantification) and sensitivity (through lower detection limits) for liquid biopsy assays. This is critical for early cancer detection, where target RNA molecules may be vanishingly scarce. It is worth noting that emerging isothermal amplification methods and CRISPR-based assays are also being explored to detect exRNAs without conventional PCR thermocycling. CRISPR Cas13-based diagnostic systems, for example, can amplify and detect specific RNA sequences with attomolar sensitivity, offering a fast and specific readout [90]. Such methods are still being refined but represent the next wave of nucleic acid detection beyond qRT-PCR/ddPCR, potentially boosting liquid biopsy sensitivity even further.

### 2.4. Other Technical Considerations

Liquid biopsy is a minimally invasive method that measures molecular products released from cells, such as proteins, cfDNAs, miRNAs and EVs. The accuracy of the results critically depends on the quality of sample collection, handling and processing. Plasma, or alternatively serum, is the preferred body fluid for liquid biopsy, and EDTA is commonly used as the anticoagulant due to its RNA-preserving properties, while heparin is generally avoided as it can inhibit downstream PCR reactions [91]. Post-collection, samples are typically stored at −80 °C to preserve their molecular content and morphology [92].

After sample acquisition, EVs must be isolated since they coexist with other extracellular components, yet there is no universal standard for isolation and analysis, posing a significant limitation [93]. Among the various isolation techniques, ultracentrifugation is the most frequently used, offering advantages such as the ability to process large fluid volumes and minimal need for additional reagents [94]. It also allows discrimination of particles based on density, shape and size [95]. Other isolation methods include size-based approaches like sequential filtration and size-exclusion chromatography, as well as capture-based, polymer-based and microfluidic technologies [96]. Once isolated, EVs require characterization and quantification, which remain technically challenging. Common methods include immunoblotting for protein markers, transmission electron microscopy and nanoparticle tracking analysis [97], while exRNA quantification typically relies on spectrophotometry and fluorimetry [98].

In the context of RNA-seq analysis, although there is no universally accepted pipeline, standard steps include quality control, raw read acquisition, alignment, transcript quantification and integration with statistical tools [99]. Quality control is often performed using quality scores in FASTQ files, summarized by tools such as FastQC [100,101]. For read alignment, HISAT2 and STAR are two of the most widely used tools due to their computational speed and ability to identify splice junctions [102]. Downstream of alignment, RNA-seq data analysis faces numerous challenges, leading to the development of various pipelines. Alignment-based approaches include Tophat-Cufflinks, Tophat-HTSeq, and STAR-HTSeq, while pseudoalignment tools like Salmon and Kallisto provide faster alternatives, especially for large datasets [103,104]. To explore biological relevance, enrichment analysis tools such as g:Profiler are employed, offering updated annotations and user-friendly interfaces [105]. Furthermore, the Genome Analysis Toolkit (GATK) serves as a gold standard for variant discovery, with its validated per-sample workflow being widely used for RNA-seq variant calling [106].

## 3. ExRNAs in MASLD and MASH

### 3.1. Correlation with Disease Severity and Progression

The identification of specific extracellular RNA biomarkers has opened new frontiers in the diagnosis and monitoring of MASLD and MASH. Emerging evidence suggests that these biomarkers, particularly certain miRNAs, not only correlate with disease severity but also aid in monitoring disease progression and assessing treatment efficacy. Multiple studies have shown that circulating exRNAs correlate with the histological severity of MASLD and progression to MASH. On this context, hepatocyte-derived miR-122-5p, constitute 72% of circulating exRNAs in MASLD patients [107]. miR-122 is considered one of the most important biomarkers for MASLD, since it correlates with hepatic steatosis severity and MASH in human and animal studies. Other miRNAs, such as miR-22, appear to promote obesity and hepatic steatosis, since their knockout in animal models has demonstrated protection against both conditions [108,109]. This might imply that particular miRNA increases are directly correlated with liver injury and inflammation, while others appear to confer protection with their levels probably reflecting development of a resistance status, in a way resembling leptin and fibroblast growth factor-21 (FGF-21) [21,110].

Accordingly, an unbiased profiling of serum exosomal miRNAs in biopsy-proven MASLD found dozens of miRNAs whose levels tracked with steatosis, inflammation, ballooning, and composite NAFLD Activity Score (NAS). Notably, miR-7151-5p levels were positively associated with overall NAS, such that high miR-7151 predicted severe disease (NAS ≥ 6) [111]. Intracellular and circulating levels of certain miRNAs often show inverse relationships. For example, miR-122—essential for lipid biosynthesis [112]—is downregulated in MASH liver tissue, while it has been shown to rise in circulation with positive correlations with histologic grades of inflammation and fibrosis [30]. In fact, miR-122 serum levels increase progressively from healthy to simple steatosis to MASH, reflecting escalating hepatocyte injury [30]. This pattern is also seen in animal studies. In MASLD mouse models, miR-122 and miR-192 were significantly elevated in circulating EVs, but reduced in liver tissue compared to controls. Their levels increased in EVs over time, suggesting active release following hepatocyte destruction. These findings were replicated in high-fat diet-induced MASLD mice, reinforcing the hypothesis that miRNAs are likely secreted into circulation as a response to hepatocyte injury [113]. Notably, in another study involving 84 serum biomarkers, circulating miR-122 levels were found to be more than seven times higher in subjects with MASH, compared to controls, and three times higher in MASH than in those with simple steatosis. Other miRNAs, including miR-192, miR-125 and miR-375—a key regulator of glucose homeostasis—exhibited at least a two-fold increase in MASH patients relatively to normal subjects [31]. Similarly, in another study with a large group of Japanese individuals attending health examinations, increased serum levels of miR-122, miR-21, miR-34a and miR-451 were observed in subjects with MASLD. Among these, only miR-122 was found to correlate with the severity of liver steatosis [29]. Lastly, a combined analysis of serum miRNAs in individuals with biopsy-confirmed MASLD or MASH, revealed that, among 26 miRNAs significantly elevated in MASH, only four (miR-21-5p, miR-151a-3p, miR-192-5p, and miR-4449) demonstrated satisfactory diagnostic potential, when combined [114].

On the other hand, conflicting results were reported from a cross-sectional study with ultrasound-proven MASLD individuals, when analysis of serum miR-20a and miR27a, but not miR-126 were found to be significantly lower in MASLD compared to healthy individuals, with further analysis showing significant inverse relationship between their levels and severe MASLD [115].

In regard to liver fibrosis, certain exRNAs have been correlated with its stage, indicating progression toward cirrhosis. A 2021 study identified three tRNA-derived small RNAs (tsRNAs)—tRF-Val-CAC-005, tiRNA-His-GTG-001, and tRF-Ala-CGC-006—that were significantly elevated in the plasma of MASLD patients and whose levels increased with higher NAS and fibrosis stage. These tsRNAs were not only present in the human blood but also rose in mouse MASH models in step with worsening collagen deposition [45] (Table 3).

LncRNAs in circulation have similarly shown correlation with disease severity. For instance, NEAT1, MEG3, and MALAT1 lncRNA levels are higher in MASLD patients, with NEAT1 upregulated in both inflammatory MASLD and advanced fibrosis [37]. Plasma NEAT1 in peripheral blood has demonstrated good diagnostic yield for MASLD (AUC ≈ 0.82), although its gradation across MASLD stages remains under investigation [38]. Another lncRNA, HULC, was reported to increase in patients with significant fibrosis [39]. Even certain circRNAs change with progression: the mitochondria-localized circRNA SCAR (identified as hsa_circ_0089763) is progressively down-regulated from simple steatosis to MASH-related cirrhosis. This decline is thought to remove a brake on metabolic inflammation, as circRNA SCAR normally helps restrain inflammasome activation in hepatocytes [48]. Taken together, a broad range of exRNA species (miRNAs, tsRNAs, lncRNAs, circRNAs) in biofluids reflect the spectrum of MASLD severity, rising or falling in concert with key pathological features like fat load, cell injury, inflammation, and fibrogenesis.

Beyond static severity, emerging evidence links exRNA dynamics to disease progression. In longitudinal studies, changes in circulating miR-122 have mirrored histological change: in one Japanese cohort, patients who achieved histologic improvement on repeat liver biopsy showed significantly reduced serum miR-122 levels compared to baseline. Conversely, persistently high or increasing miR-122 over time has been associated with ongoing liver injury and even a higher risk of progression to HCC [116]. These findings suggest that serial exRNA measurements could potentially monitor disease trajectory. Similarly, tsRNAs including tRF-Val-CAC-005, tiRNA-His-GTG-001, and tRF-Ala-CGC-006 were increased in those MASLD patients with more advanced fibrosis, hinting that they might predict fibrotic progression risk [45]. However, prospective validation is needed. Altogether, the current literature supports that exRNAs correlate strongly with MASLD/MASH severity and may provide insight into disease progression risk, complementing traditional methods and markers.

### 3.2. exRNA Changes with Treatment Response

Early data indicates that certain exRNAs also respond to therapeutic interventions, paralleling clinical improvement. Weight loss—the cornerstone of MASLD/MASH management—appears to normalize some elevated miRNAs. In a pilot study of diet/exercise intervention, patients who achieved resolution of MASH on follow-up biopsy had significant declines in circulating miR-122 levels [117]. This makes biological sense, as miR-122 release into blood is tied to hepatocyte injury; successful therapy that heals the liver reduces this leakage. Similarly, miR-34a and miR-21, which are elevated in active MASH, have been observed to decrease after sustained weight loss or bariatric surgery in small case series (in line with reduced inflammation), though larger studies are needed for confirmation [118,119].

Not all exRNAs may normalize with therapy, as some of them reflect irreversible fibrosis rather than reversible injury; however, those tied to ongoing pathogenic processes (i.e., ballooning and inflammation) show promise as pharmacodynamic markers. For example, experimental MASH treatments that target fibrogenic pathways have been noted to alter exRNA profiles in animal models: an antifibrotic drug reduced miR-21-5p (a pro-fibrotic miRNA) in rodent MASH models alongside improvement in fibrosis [120]. These findings hint that exRNAs could serve as liquid biopsy readouts of treatment response, decreasing as the liver heals. Such exRNA biomarkers of response would be especially valuable in clinical trials to gauge drug efficacy without repeated biopsies [111].

### 3.3. Diagnostic Accuracy of exRNAs Versus Traditional Biomarkers

A major appeal of exRNA biomarkers is their potential to improve non-invasive diagnosis of MASLD and MASH, compared to conventional tests like alanine aminotransferase (ALT), FIB-4 score or imaging. Traditional serum markers have limitations: ALT is neither sensitive nor specific as its levels can be normal in many MASLD patients even with advanced disease, and indices like FIB-4 are useful for fibrosis but not designed to detect MASH. In contrast, specific exRNA signatures show high diagnostic accuracy. Multi-miRNA panels in particular have yielded promising results in distinguishing MASH from simple steatosis. For example, it has been suggested that combining three serum miRNAs (miR-122, miR-192, and miR-21) can non-invasively identify MASH with performance comparable to specialized protein biomarkers. In that biopsy-proven MASLD cohort, the aforementioned miRNA panel achieved an area under the receiver operating characteristic curve (AUROC) of about 0.81 for differentiating MASH from steatosis, essentially matching the AUROC of cytokeratin-18 (CK-18) M30 fragments—0.81—and outperforming ALT alone (AUROC~0.77) [30]. The panel’s sensitivity and specificity were high (91% and 83% at optimal cutoff) and improved further when combined with CK-18 levels. Another large study identified a four-miRNA serum signature—miR-122-5p, miR-1290, miR-27b-3p, and miR-192-5p—that discriminated MASLD patients from healthy controls with AUC~0.89, significantly better than ALT or FIB-4 (which had AUC~0.79 each) [121]. Notably, the miRNA panel maintained high accuracy across mild (NAS < 3) and severe (NAS ≥ 5) MASLD, indicating robustness across disease stages [121]. These data suggests exRNA panels can detect MASLD even when standard tests are borderline. In terms of single markers, several outperform traditional labs as diagnostic tools. For example, plasma lncRNA liver X receptor-induced sequence (LeXis) was recently proposed as a MASH-specific marker. LeXis levels were found to be significantly higher in patients with MASH compared to those without. Using an optimal cutoff, LeXis could diagnose MASH with 67% sensitivity and 100% specificity, far exceeding the specificity of ALT or ultrasound which often misclassify other causes [41]. Such high specificity in that small cohort underscores the unique disease-associated signal carried by some ncRNAs, although it should be taken with a grain of salt since regression analyses in small datasets tend to overfit. Another study reported that blood levels of lncRNA MALAT1 and HULC rise with advanced fibrosis, suggesting they might complement FIB-4 in identifying high-risk MASLD patients [39,122,123]. In summary, specific exRNA signatures—alone or in combination—show equal or superior diagnostic performance to conventional blood tests for MASLD and MASH. By capturing multiple facets of the disease, including steatosis, injury and fibrosis in one readout, exRNA-based assays could substantially improve non-invasive diagnosis and reduce reliance on liver biopsy.

### 3.4. Mechanistic Roles of exRNA in MASLD Pathophysiology

Beyond their diagnostic utility, exRNAs are also mechanistic mediators in MASLD and MASH, actively contributing to hepatocyte injury, lipid dysregulation and fibrosis. Many pathogenic processes in these conditions are driven or modulated by non-coding RNAs. For example, the aforementioned miR-122 plays a central role in hepatic lipid metabolism: it promotes healthy hepatocyte differentiation and represses lipogenic genes [124,125]. In MASH livers, miR-122 expression is often downregulated in the tissue even as its levels rise in the circulation due to leakage and this loss of miR-122 derepresses its targets, leading to exacerbated lipogenesis and fibrosis [126,127,128]. Physiologically, miR-122 appears to regulate proinflammatory, cell-proliferating, and morphology-disrupting genes of the chemokines, growth factors and metalloprotease family, including C–C motif chemokine ligand 2 (CCL2), CCL3, insulin-like growth factor receptor 1 (IGFR1), matrix metallopeptidase 8 (MMP8) and MMP9, thereby maintaining hepatic homeostasis [127]. Experimentally, deleting miR-122 in mice causes steatosis and steatohepatitis, whereas restoring miR-122 alleviates lipid accumulation [124,125]. Another key player is miR-34a, which is upregulated by fatty acids and oxidative stress; miR-34a impairs mitochondrial function and increases hepatocyte apoptosis by silencing SIRT1, thereby aggravating liver injury in MASLD [129,130]. miR-21 is a pro-fibrotic miRNA induced in MASH and targets multiple antifibrotic genes, including transforming growth factor (TGF)-β regulators, skewing the balance toward collagen deposition [120,131,132]. Inhibiting miR-21 in MASH models attenuates stellate cell activation and fibrosis, underscoring its mechanistic role [120,133]. Similarly, miR-221/222 are linked to hepatocyte ballooning and inhibit cell cycle regulators [134,135], and miR-223, a molecule released largely from inflammatory cells, modulates the inflammatory response in MASH [136,137]. Interestingly, many of these pathogenic miRNAs are exported in EVs and can act on distant cells. For instance, adipocyte-derived exosomal miR-122 can enter hepatocytes and contribute to MASLD by modulating Sirt1 signaling, whereas its hepatocyte-derived counterpart can function paracrinally to regulate the behavior of Kupffer cells, inducing their proinflammatory M1 phenotype [138,139]. This crosstalk suggests exRNAs are not just byproducts of liver injury but inter-organ messengers that amplify metabolic dysfunction systemically. LncRNAs and cRNAs also have direct pathogenic functions. NEAT1, a lncRNA highly expressed in hepatocytes, has been shown to promote hepatic fat accumulation by sequestering a miR-212-5p/GRIA3 regulatory axis, thereby enhancing lipogenic signaling [40]. NEAT1 is also induced by inflammatory stimuli and may facilitate the transition from simple steatosis to steatohepatitis [38,140]. Another lncRNA, MALAT1, is implicated in fibrosis: MALAT1 upregulation in hepatocytes and stellate cells can drive epithelial–mesenchymal transition and collagen gene expression—a finding consistent with the observation that MALAT1 levels are higher in fibrotic MASLD [123,141,142]. On the other hand, some lncRNAs have protective roles; for example, LeXis normally helps cholesterol efflux and its higher circulating level in MASH might reflect a compensatory response to lipid overload [41,143]. CircRNA SCAR, which resides in mitochondria, was discovered to blunt reactive oxygen species (ROS) production by binding mitochondrial ATP5B; in MASH, loss of circSCAR leads to excess mitochondrial ROS and “metaflammation,” exacerbating steatohepatitis [48]. Even the novel tsRNAs appear to have mechanistic impact: the tRF-3001b fragment, elevated in MASLD mice, directly targets the 5′ UTR of *Prkaa1* (AMPKα) mRNA, reducing AMPK levels and thus impairing autophagy, which in turn promotes lipid accumulation. Silencing tRF-3001b in MASLD models restored autophagy and improved liver histology [144].

In summary, it appears that exRNAs are closely involved in MASLD and MASH pathophysiology, by regulating four major gene networks: lipid metabolism, apoptosis, inflammation, and fibrogenesis. This mechanistic insight opens the door to therapeutic modulation of exRNAs (e.g., anti-miR therapies) to treat MASLD/MASH at the molecular level. ExRNAs not only reflect the disease state but actively shape it by mediating the cross-talk between injured hepatocytes, stellate cells, and immune cells that drives progression from steatosis to steatohepatitis and fibrosis.

## 4. Current Status of Liquid Biopsy in MASLD and MASH Management

Liquid biopsy has emerged as a promising alternative to traditional liver biopsy, which still remains the gold standard for diagnosing liver disease and assessing liver fibrosis severity. Liver biopsy involves the extraction of a small tissue sample from the liver for microscopic examination, but it is an invasive procedure fraught with limitations, including patient eligibility criteria and associated risks from the intervention. In fact, many patients who undergo liver biopsy as part of clinical studies do not necessarily meet the eligibility criteria, thus indicating lack of better pre-biopsy strategies that can more accurately target appropriate candidates.

As a non-invasive diagnostic tool, liquid biopsy presents a more accessible and with lower risk alternative for the patients. One of its main advantages is the ability to analyze circulating biomarkers, including miRNAs, which are present in bodily fluids like serum. These biomarkers can provide valuable insights into the disease diagnosis and progression, without the need of tissue extraction. Utility and applications of different miRNA molecule measurements were discussed earlier in this review [29,31,37,45,112,114,115,121,145,146,147].

In regard to serum biomarkers, many of them have been associated with the pathogenesis of MASLD and MASH, as well as liver fibrosis. However, their clinical utility is constrained by low specificity and sensitivity. Moreover, these biomarkers often fail to accurately predict disease severity or fibrosis stage and, relatively, even MASLD scores themselves, including assessment for liver fibrosis, can be affected by individual factors, such as obesity [148]. For instance, measuring circulating monocyte perilipin-2 (PLIN2) levels has shown promising results in diagnosing MASH, while RAB14 levels have been found to be useful in predicting liver fibrosis. These biomarkers have demonstrated the potential to outperform traditional markers and scores, such as FIB-4 score, MASLD fibrosis score, and the AST-to-platelet ratio, as far as sensitivity and specificity are concerned [149].

Additional biomarkers, including leptin [21] and FGF-21 [110] have also been explored as potential tools for MASLD and MASH diagnosis and progression. Both have been shown to be positively associated with MASLD and MASH and have been proposed as possible disease markers. However, further studies are needed to further clarify the accuracy of such serum biomarkers.

Another innovative approach involves the use of modified aptamer proteomics, a technique that enables the high-throughput analysis of thousands of proteins in a single sample. Particularly, a study assessed over 5000 proteins in subjects with histologically confirmed MASLD and validated protein-phenotype models, which can reliably assess various stages of the disease, including steatosis, hepatocellular ballooning, lobular inflammation and fibrosis. Findings were comparable to established biomarkers and traditional methods of MASLD assessment, further supporting the potential of liquid biopsy as a non-invasive diagnostic tool in liver disease [150].

While these findings are encouraging, it is important to note that additional validation studies across diverse populations are necessary. Differences in genetic background, environmental factors, and comorbid conditions can influence biomarker levels and limit generalizability. Therefore, broader research is required to confirm the diagnostic performance of these novel molecules and to ensure their applicability in various clinical settings worldwide.

## 5. Comparison of Liquid Biopsy with Traditional Diagnostic Methods

MASLD spectrum diagnosis requires fat presence of more than 5% in the liver [151]. A variety of methods are available to assess the presence of liver steatosis, each one with specific limitations and advantages, as detailed in Table 4. Magnetic resonance spectroscopy (MRS) is a fairly accurate method that quantifies the proton density fat fraction (PDFF), providing a precise measurement of liver fat, although it is a relatively limited modality, requiring specialized knowledge and is not routinely available in everyday clinical practice. For this reason, MRI-PDFF has been developed as a more accessible method, avoiding patient-related confounders such as body mass index (BMI) and comorbidities. MRI-PDFF does not require spectroscopy and can be performed on routine scanners, making it a more feasible option in clinical settings. Nevertheless, MRI-PDFF is not without limitations, including claustrophobia, and the presence of metal implants, which can hinder its utility [152,153].

When it comes to liver fibrosis, current imaging techniques lack the ability to directly detect molecular signatures of fibrosis. Instead, the proposed methods aim to indirectly assess fibrosis presence by using biomarkers related to liver parameters, including liver stiffness, metabolites and image texture. Among these, liver stiffness is considered the leading marker, correlating with the collagen deposition on the liver [159,160].

Transient elastography (TE) is particularly effective in assessing advanced fibrosis and it serves as a valuable prognostic tool. This study as other ultrasound-based tests, even that they are widely accessible, they are limited in subjects with ascites or obesity [159]. In contrast, Magnetic Resonance Elastography (MRE) and MRI-PDFF have shown superior diagnostic accuracy for steatosis and fibrosis, outperforming TE in certain contexts [161], and also indicating more reliably the liver stiffness [156]. Additionally, two-dimensional shear wave elastography has demonstrated potential for early fibrosis detection in chronic liver disease [162]. Acoustic radial force imaging has also been explored, but it has been outperformed by MRE in assessing liver fibrosis [163].

Other traditional diagnostic methods often rely on serum markers such as ALT and platelet count. ALT, in particular, has demonstrated a good negative predictive value (NPV), with 11% of subjects with normal ALT having eventually MASH. On the other hand, an ALT level two times greater than the upper limit of normal showed 50% sensitivity and 61% specificity for detecting MASH [164].

Secondly, the MASH test takes into consideration individual characteristics including age and BMI, serum aminotransferases and lipids as well as other serum markers, with a sensitivity of just 33% but a specificity of 94% [165].

As liver fibrosis progresses toward advanced fibrosis or cirrhosis, serum ALT levels typically decline, while aspartate aminotransferase (AST) levels remain stable or increase, leading to a higher AST/ALT ratio. Among the various scoring systems, the MASLD fibrosis core, formerly known as NAFLD fibrosis score (NFS), is notable for its comprehensive approach, incorporating individual factors, including age, BMI, aminotransferase levels, glucose, platelet count and albumin. Using a high cutoff of more than 0.676, was related with a positive predictive value (PPV) of 82% for advanced F3–F4 fibrosis, with sensitivity of 43% and specificity of 96%. On the other hand, a low cutoff value, of less than −1.455, had a NPV of 88%, with a sensitivity of 77% and a specificity of 71% [166].

Another very useful tool, being used more frequently in daily clinical practice lastly, is the FIB-4 score, which combines laboratory tests, including platelet count, AST, ALT with age. This score has shown promise in predicting outcomes in MASLD patients and has been shown to be superior to other serologic markers [157]. A FIB-4 score < 1.3 has a 90% NPV for stage 3–4 fibrosis, whereas a score of >2.67 has a PPV of 75–100%. However, its specificity declines with age, with studies showing that age-adjusted lower cutoffs are needed in patients of more than 65 years of age. It should also be used with caution in ages of <35, since it performed poorly in diagnosing advanced liver fibrosis in that age group [167].

Finally, the AST-to-platelet ratio index (APRI), which is calculated using the AST level and platelet count, has shown utility in predicting liver outcomes, including patients with MASLD. This index has primarily been studied in patients with Hepatitis C virus (HCV) infection, human immunodeficiency virus (HIV) and HCV coinfection and alcohol-related liver disease. In one retrospective series involving 320 patients, APRI was found to be effective in predicting liver-related outcomes, including death and liver transplantation [158].

Having said that, there is ongoing evidence that liquid biopsy can offer significant benefits in less invasively diagnosing MASLD or MASH and serve as an important alternative of the traditional diagnostic methods. For example, in a study involving 41 biopsy-confirmed MASLD patients, serum exRNAs correlated with steatosis, inflammation, ballooning, NAS, and fibrosis, suggesting staging and prognostic utility of exRNAs. Of note, miR-133b, miR-4436a, miR-4709-3p, and miR-8079 were positively associated with liver enzymes elevations [111]. Accordingly, in a study including 228 biopsy-proven MASLD patients, an EV miRNA signature (miR-27b-3p, miR-30a-5p, miR-122-5p, miR-375-3p, miR-103a-3p, let-7d-5p, let-7f-5p) discriminated steatohepatitis and significant fibrosis (F ≥ 2), thus highlighting the potential diagnostic and pathophysiologic relevance in “at-risk MASH” [168].

## 6. Future Perspectives for exRNA Diagnostics in MASLD and MASH

The field of miRNA-based targeted therapies continues to evolve in recent years, with significant strides being made in treatment of liver conditions, including MASLD, MASH and hepatocellular carcinoma. As discussed earlier, miRNAs have a great potential for diagnostics, as well as for pharmaceutical utilities, however this requires a deep understanding of their physiology and pathophysiology, so that carrier molecules are developed and effectively store the sensitive genetic information included in the miRNAs. This way, they will be able to “travel” in the bloodstream, until the target-organ is reached and the treatment is delivered appropriately.

Recent advances in drug delivery systems, such as lipid-based nanoparticles, liposomes, and multifunctional envelope nanodevices (MENDs), are helping to address the issue of poor cellular uptake and bioavailability, showing promising results [109,169,170]. Moreover, novel strategies propose the use of EVs as miRNA carriers, with encouraging results in preclinical studies, demonstrating efficient delivery of therapeutic miRNAs and even tumor suppression in liver cancer cells [171].

Additionally, mimicking is another mechanism that holds great promise for future therapeutic interventions in liver diseases, as it can prevent disease progression, by specific miRNA targeting, since it has demonstrated the ability to decrease relevant circulating miRNAs levels in the bloodstream [172].

On the other hand, while polyethylene glycol attachment to biochemical molecules, process known as PEGylation, has been widely used to stabilize nanoparticles and prolong their circulation time, it might often impair cellular uptake and tissue delivery [173]. Recent developments, such as the pH-sensitive cationic lipid YSK05 in MENDs, have successfully improved delivery and endosomal escape, enhancing therapeutic efficacy [170]. Accordingly, polymeric vectors like jetPEI are under investigation [174], but their safety and efficacy remain to be fully validated.

While this review focuses on the diagnostic utility of exRNAs, particularly miRNAs, it is worth noting that several miRNAs implicated in MASLD, such as miR-122 and miR-21, are also under investigation as potential therapeutic targets [107,175]. Antagomirs, locked nucleic acid (LNA) inhibitors, and miRNA mimics have shown promise in preclinical models, demonstrating the potential to reduce hepatic steatosis, inflammation, and fibrosis [176]. However, clinical translation remains limited due to barriers such as off-target effects, delivery system inefficiencies, and immune-related concerns. Further advances in delivery technologies and safety profiling will be essential for realizing the therapeutic potential of miRNAs in MASLD.

## 7. Challenges and Limitations

Despite the growing promise of EVs and exRNAs as biomarkers for liver diseases, several significant limitations currently delay their broader clinical translation and adoption. One of the greatest challenges is the lack of standardized methodologies. Consistent and reproducible results depend heavily on well-defined protocols for sample collection, timely processing, storage, and RNA extraction. However, variations in critical steps, such as the choice of anticoagulant (e.g., EDTA vs. heparin), delays in plasma separation, and inconsistent freezing or thawing practices, can significantly affect RNA yield and integrity [91]. Differences in RNA isolation techniques, including column-based vs. organic extraction methods or EV enrichment approaches, lead to variability in both RNA quantity and subtype representation [177]. Even the choice of EV isolation method (e.g., ultracentrifugation vs. size-exclusion chromatography) influences downstream RNA profiles [178]. These inconsistencies are compounded by assay-level variability across platforms such as qRT-PCR, ddPCR, and RNA-seq, each with unique sensitivities, normalization strategies, and susceptibility to technical artifacts. Contamination, improper storage, and differing equipment or reagent use further exacerbate reproducibility issues [179]. Additionally, biological heterogeneity among patients, such as disease stage, comorbidities, or medication status, contributes to inter-individual variability in exRNA profiles, which complicates even further the establishment of reproducible universal molecular signatures [179].

Technically, RNA quantification from EVs presents a set of challenges that are yet to be fully resolved. One of the primary difficulties is the low abundance of exRNAs in body fluids such as plasma or urine, which often necessitates the use of large sample volumes or specialized enrichment protocols. Moreover, exRNAs are inherently heterogeneous in size and sequence and are highly susceptible to degradation by RNases. The process of isolating these RNAs from complex biological fluids requires advanced methodologies, such as ultracentrifugation, size-exclusion chromatography, or immunoaffinity capture, that are not only labor-intensive but also prone to variability. Additionally, quantification techniques like NanoDrop™ and Qubit™ suffer from issues related to sensitivity and specificity, while more advanced methods such as qRT-PCR and ddPCR can be affected by technical artifacts, including primer dimer formation and genomic contamination. Secondary RNA structures can also interfere with detection accuracy, necessitating careful handling to preserve data quality.

Biological and analytical confounders further complicate the interpretation of liquid biopsy data. Endogenous molecules such as anti-mRNAs and artificial miRNA mimics can alter the activity of specific miRNAs, thus skewing assay results. Detection sensitivity and specificity also vary across platforms, such as PCR, microarrays, and sequencing, leading to discrepancies in exRNA profiles depending on the analytical approach used. Methods like size-exclusion chromatography can introduce selection bias by isolating EVs within a specific size range, potentially excluding biologically relevant subpopulations. Furthermore, experimental conditions, such as the use of serum-containing media in EV-producing cell cultures, can introduce non-EV contaminants, making it difficult to interpret results accurately. The use of different filter membrane types, protein quantification kits, and particle measurement techniques can also affect reproducibility and recovery efficiency across laboratories.

A final and critical limitation lies in the underreporting of methodological details in the current literature. Many studies fail to comprehensively document the specific reagents, protocols, or analytical parameters used, impeding reproducibility and comparative evaluation. This lack of transparency, combined with high inter-sample and inter-study variability in exRNA profiles, even among samples from the same disease type, undermines the reliability of biomarker discovery efforts. Without large-scale validation in diverse patient populations and rigorous standardization of analytical workflows, the clinical utility of EV-derived exRNAs as liver disease biomarkers will remain limited.

## 8. Conclusions

The emerging role of exRNAs in liquid biopsy represents a promising advancement in the diagnosis, monitoring, and management of MASLD and its more advanced form, MASH. The detection of exRNAs, particularly miRNAs and circulating EVs offers a noninvasive and highly sensitive approach to assessing disease progression, identify fibrosis stages and monitor treatment responses. Key biomarkers, such as miR-122, miR-34a and miR-21, show significant potential in reflecting the molecular and histological changes associated with MASLD and MASH. Several challenges remain in fully integrating exRNA-based liquid biopsy into clinical practice. Clinical translation of exRNA findings into routine medical practice faces obstacles related to cost, accessibility, and integration with existing diagnostic tools. Addressing these limitations is critical to unlocking the full clinical potential of exRNA biomarkers, including their ability to enable earlier, less invasive diagnosis, improve risk stratification, and guide personalized treatment strategies in MASLD.

Future research should prioritize improving the sensitivity and specificity of liquid biopsy markers while working toward establishing exRNA-based diagnostics as a reliable, noninvasive screening tool for early detection and precise monitoring of MASLD and MASH. Advancing these technologies will contribute to personalized medicine, improving patient outcomes by tailoring treatment based on individual molecular profiles. It is anticipated that in the near future, MASLD diagnosis and treatment will be increasingly guided by each patient’s unique molecular signature. This is possible because each patient exhibits a unique molecular signature, including specific exRNA profiles and histone modifications, which reflect their individual disease mechanisms and progression patterns. Applying these personalized molecular insights can help tailor interventions to target the underlying pathophysiology in each case, improving treatment efficacy and patient outcomes. Continued evaluation of liquid biopsy technologies, combined with a deeper understanding of the molecular mechanisms underlying MASLD, holds great promise for revolutionizing liver disease management.

## Figures and Tables

**Table 1:** Overview of different types of extracellular RNAs and their functions.

Types of exRNA	Description	Function	Relevance to MASLD	Relevant Studies
miRNA	Small non-coding RNA molecules (19–25 nucleotides)	Regulates gene expression post-transcription; involved in lipid synthesis in liver parenchymal cells [28]	miR-122, miR-34a, miR-21, etc., are potential biomarkers for MASLD [3,19,20,23,24,29]	In vivo studies showed elevations in serum and EVs in MASLD patients and high-fat diet mouse models; inversely downregulated in liver tissue; correlates with NAS and fibrosis [29,30,31].
mRNA	Messenger RNA that carries genetic information from DNA [32]	Provides instructions for protein synthesis [32]	Found in EVs; can reflect liver function [33]	In vitro studies revealed new biomarkers for hepatic fibrosis [34].
lncRNA	Non-coding RNA (>200 nucleotides) [35]	Does not encode proteins. Involved in gene expression at a transcriptional and post-transcriptional stage [36]	Early diagnosis and progression of MASLD [37,38,39]	Some of them promote liver fat buildup, while others may help remove cholesterol or reflect the body’s response to fat overload; others are linked to liver fibrosis [37,39,40,41].
siRNA	Small interfering RNA	Regulates expression of genes via RNA interference and degradation of specific mRNAs at a post-transcription level [42]	Participates in liver protein synthesis [42]	In vitro studies showed great diagnostic and therapeutic value in MASLD [43].
tRNA	Transfer RNA molecule involved in protein synthesis	Facilitates translation of mRNA into proteins [44]	Participates in liver protein synthesis [45]	In vivo studies showed plasma elevations in MASLD, with higher levels in higher NAS and fibrosis stage; same in mouse models of MASH [45].
circRNA	Circular RNA molecule, stable and covalently closed [46]	Modulates transcription and translation processes [47]	Challenging to be evaluated in research due to its structure	Down-regulated when progressing from simple steatosis to MASH-related cirrhosis in in vitro studies [48].

Abbreviations: circRNA, circular RNA; EV, extracellular vesicles; exRNA, extracellular RNA; lncRNA, long non-coding; MASLD, metabolic dysfunction-associated steatotic liver disease; MASH: metabolic dysfunction-associated steatohepatitis; miRNA, microRNA; mRNA, messenger RNA; NAS, non-alcoholic fatty liver disease activity score; siRNA, small interfering RNA; tRNA, transfer RNA.

**Table 2:** Summary of studies describing the exRNA Detection and Analysis Methods.

Method	Key Feature of the Study	Relevance
SILVER-seq	RNA-seq from a single drop fo serum	Enables whole-transcriptome exRNA profiling from ultra-low input volumes.
PALM-Seq	Adds poly(A) tails to non-polyadenylated exRNAs	Captures a broader exRNA range (e.g., miRNAs, tRNAs).
NGS for exRNA	Allows analysis in cell-free mRNAs	Used for discovery of novel biomarkers and cancer monitoring.
exceRpt pipeline/exRNA Atlas	Bioinformatics tools for exRNA data analysis	Helps filter contaminants and manage fragmented plasma RNA.
Single-cell RNA-seq (scRNA-seq)	Sequencing transcripts in individual cells	Complements exRNA data by identifying the source cells of extracellular signals.
Single-EV sequencing	RNA-seq from individual extracellular vesicles	Helps profiling RNAs from single exosomes or microvesicles.
qRT-PCR	Traditional RNA quantification technique	Validates candidate exRNAs but limited in sensitivity for low-abundance targets.
Droplet digital PCR (ddPCR)	Partitions samples into droplets for high-sensitivity detection	Better precision and reproducibility for serum miRNA quantification compared to qRT-PCR.

Abbreviations: EV, extracellular vesicles; exRNA, extracellular RNA; miRNA, microRNA; qRT-PCR, quantitative reverse transcription polymerase chain reaction; RNA-seq, RNA sequencing; tRNA, transfer RNA.

**Table 3:** Key RNA Biomarkers for MASLD and MASH.

RNA Subtype	Expression Pattern	Clinical Relevance
miR-122	Increased in MASLD and MASH	Strong diagnostic marker for MASLD and liver dysfunction; correlates with severity of hepatic steatosis and with MASH Positively related to MASLD in animal studies [113].
miR-22-3p	Increased in MASLD and MASH	Its “knockout” protects against obesity and hepatic steatosis in mice [108]
miR-21	Increased in MASLD and MASH	Diagnostic potential for MASLD and MASH, indicating disease progression, especially when combined with biomarkers [109]
miR-192-5p	Increased in MASLD and MASH	Diagnostic potential for MASLD and MASH, indicating disease progression, especially when combined with biomarkers [114] miR-192 also positively related to MASLD in animal studies [113].
tRNAs (tRF-Val-CAC-005, tiRNA-His-GTG-001, tRF-Ala-CGC-006)	Increased in MASLD	Significantly elevated levels in MASLD compared to non-MASLD; levels associated with MASLD activity score; findings confirmed in mouse model [45]

Abbreviations: MASLD, metabolic dysfunction-associated steatotic liver disease; MASH, metabolic dysfunction-associated steatohepatitis; miR, micro-RNA; tRNA, transfer RNA.

**Table 4:** Comparison of Liquid Biopsy and Traditional Diagnostic Methods for MASLD.

Diagnostic Method	Advantages	Limitations	Applications in MASLD/Fibrosis
Liquid biopsy [biomarkers, exRNAs (miRNAs, mRNAs, etc.)]	Non-invasive, repeatable	Not available in routine daily practice as of now; molecular signature of each individual can affect interpretation of results	Emerging tool for diagnosis, monitoring and staging of MASLD and MASH
Liver biopsy	Gold standard; accurate for fibrosis staging and diagnosis	Invasive, can have complications; limited by patient eligibility criteria	Still the definitive method for accurate diagnosis [154]
Ultrasound-based elastography/transient elastography	Widely accessible, non-invasive, low-cost	Less accurate for early-stage fibrosis, affected by obesity and ascites	Can detect liver stiffness attributed to liver fibrosis [155]
MRI-based elastography	Non-invasive, high diagnostic accuracy in fibrosis detection	Requires expensive equipment, limited availability	More effective than ultrasound-based techniques, in assessing liver parameters, including liver stiffness and fibrosis [156]
Serum markers (AST, ALT, FIB-4 score, platelets, etc.)	Non-invasive, cost-effective, widely accessible	Low sensitivity and specificity	Useful for initial screening and stratification [157,158]

Abbreviations: AST, aspartate aminotransferase; ALT, alanine aminotransferase; exRNA, extracellular RNA; MRI, magnetic resonance imaging; MASLD, metabolic dysfunction-associated steatotic liver disease; MASH, metabolic dysfunction-associated steatohepatitis, miRNA, micro-RNA; mRNA, messenger RNA.

## Data Availability

No new data were created or analyzed in this study.

## Abbreviations

ExRNAs	Extracellular RNAs
MASLD	Metabolic-Associated Steatotic Liver Disease
MASH	Metabolic-Associated Steatohepatitis
miRNA	MicroRNA
circRNA	circular RNA
EVs	Extracellular Vesicles
tRNA	Transfer RNA
tsRNA	tRNA-derived Small RNA
cfDNA	Circulating Free DNA
RNA-seq	RNA Sequencing
ALT	Alanine Aminotransferase
AST	Aspartate Aminotransferase
MRE	Magnetic Resonance Elastography
MRI-PDFF	Magnetic Resonance Imaging Proton Density Fat Fraction
TE	Transient Elastography
NFS	NAFLD (Non-Alcoholic Fatty Liver Disease) Fibrosis Score
APR	Aspartate Aminotransferase to Platelet Ratio
qRT-PCR	Quantitative Reverse Transcription Polymerase Chain Reaction
ddPCR	Droplet Digital PCR
MRS	Magnetic Resonance Spectroscopy
UV-vis	Ultraviolet–Visible Spectrophotometry
NPV	Negative Predictive Value
PPV	Positive Predictive Value

## References

[1] Chan W.-K., Chuah K.-H., Rajaram R.B., Lim L.-L., Ratnasingam J., Vethakkan S.R. (2023). Metabolic Dysfunction-Associated Steatotic Liver Disease (MASLD): A State-of-the-Art Review. J. Obes. Metab. Syndr..

[2] Younossi Z.M., Kalligeros M., Henry L. (2025). Epidemiology of metabolic dysfunction-associated steatotic liver disease. Clin. Mol. Hepatol..

[3] Sato-Espinoza K., Chotiprasidhi P., Liza E., Placido-Damian Z., Diaz-Ferrer J. (2024). Evolution of liver transplantation in the metabolic dysfunction-associated steatotic liver disease era: Tracking impact through time. World J. Transplant..

[4] Maurice J., Manousou P. (2018). Non-alcoholic fatty liver disease. Clin. Med..

[5] Castagneto-Gissey L., Bornstein S.R., Mingrone G. (2024). Can liquid biopsies for MASH help increase the penetration of metabolic surgery? A narrative review. Metabolism.

[6] Torre E., Di Matteo S., Martinotti C., Bruno G.M., Goglia U., Testino G., Rebora A., Bottaro L.C., Colombo G.L. (2024). Economic Impact of Metabolic Dysfunction-Associated Steatotic Liver Disease (MASLD) in Italy. Analysis and Perspectives. Clin. Outcomes Res..

[7] Eslam M., Sanyal A.J., George J., Sanyal A., Neuschwander-Tetri B., Tiribelli C., Kleiner D.E., Brunt E., Bugianesi E., Yki-Järvinen H. (2020). MAFLD: A consensus-driven proposed nomenclature for metabolic associated fatty liver disease. Gastroenterology.

[8] Dale K., Fallouh Y., Alkhouri N. (2024). MASLD and MASH: How a change of nomenclature may impact our approach in treating liver disease. Expert Opin. Investig. Drugs.

[9] Targher G., Byrne C.D., Tilg H. (2024). MASLD: A systemic metabolic disorder with cardiovascular and malignant complications. Gut.

[10] Zhang Y.N., Fowler K.J., Ozturk A., Potu C.K., Louie A.L., Montes V., Henderson W.C., Wang K., Andre M.P., Samir A.E. (2020). Liver fibrosis imaging: A clinical review of ultrasound and magnetic resonance elastography. J. Magn. Reson. Imaging.

[11] Zoncapè M., Liguori A., Tsochatzis E.A. (2024). Non-invasive testing and risk-stratification in patients with MASLD. Eur. J. Intern. Med..

[12] Hsu C., Caussy C., Imajo K., Chen J., Singh S., Kaulback K., Le M.-D., Hooker J., Tu X., Bettencourt R. (2018). Magnetic resonance vs transient elastography analysis of patients with non-alcoholic fatty liver disease: A systematic review and pooled analysis of individual participants. Clin. Gastroenterol. Hepatol. Off. Clin. Pract. J. Am. Gastroenterol. Assoc..

[13] Selvaraj E.A., Mózes F.E., Jayaswal A.N.A., Zafarmand M.H., Vali Y., Lee J.A., Levick C.K., Young L.A.J., Palaniyappan N., Liu C.-H. (2021). Diagnostic accuracy of elastography and magnetic resonance imaging in patients with NAFLD: A systematic review and meta-analysis. J. Hepatol..

[14] Karlas T., Weise L., Kuhn S., Krenzien F., Mehdorn M., Petroff D., Linder N., Schaudinn A., Busse H., Keim V. (2017). Correlation of cell-free DNA plasma concentration with severity of non-alcoholic fatty liver disease. J. Transl. Med..

[15] Buzova D., Braghini M.R., Bianco S.D., Re O.L., Raffaele M., Frohlich J., Kisheva A., Crudele A., Mosca A., Sartorelli M.R. (2022). Profiling of cell-free DNA methylation and histone signatures in pediatric NAFLD: A pilot study. Hepatol. Commun..

[16] Buzova D., Maugeri A., Liguori A., Napodano C., Lo Re O., Oben J., Alisi A., Gasbarrini A., Grieco A., Cerveny J. (2020). Circulating histone signature of human lean metabolic-associated fatty liver disease (MAFLD). Clin. Epigenet..

[17] Sookoian S., Pirola C.J. (2017). Cell-free DNA methylation as liquid biopsy for the assessment of fibrosis in patients with nonalcoholic steatohepatitis: A gap between innovation and implementation. Hepatobiliary Surg. Nutr..

[18] Yiğit B., Boyle M., Özler O., Erden N., Tutucu F., Hardy T., Bergmann C., Distler J.H.W., Adalı G., Dayangaç M. (2018). Plasma cell-free DNA methylation: A liquid biomarker of hepatic fibrosis. Gut.

[19] Howell L.S., Ireland L., Park B.K., Goldring C.E. (2018). MiR-122 and other microRNAs as potential circulating biomarkers of drug-induced liver injury. Expert Rev. Mol. Diagn..

[20] Mikulski D., Kościelny K., Dróżdż I., Mirocha G., Nowicki M., Misiewicz M., Perdas E., Strzałka P., Wierzbowska A., Fendler W. (2024). Serum Levels of miR-122-5p and miR-125a-5p Predict Hepatotoxicity Occurrence in Patients Undergoing Autologous Hematopoietic Stem Cell Transplantation. Int. J. Mol. Sci..

[21] Polyzos S.A., Aronis K.N., Kountouras J., Raptis D.D., Vasiloglou M.F., Mantzoros C.S. (2016). Circulating leptin in non-alcoholic fatty liver disease: A systematic review and meta-analysis. Diabetologia.

[22] Tobaruela-Resola A.L., Milagro F.I., Elorz M., Benito-Boillos A., Herrero J.I., Mogna-Peláez P., Tur J.A., Martínez J.A., Abete I., Zulet M.Á. (2024). Circulating miR-122-5p, miR-151a-3p, miR-126-5p and miR-21-5p as potential predictive biomarkers for Metabolic Dysfunction-Associated Steatotic Liver Disease assessment. J. Physiol. Biochem..

[23] Liu X.-L., Pan Q., Zhang R.-N., Shen F., Yan S.-Y., Sun C., Xu Z.-J., Chen Y.-W., Fan J.-G. (2016). Disease-specific miR-34a as diagnostic marker of non-alcoholic steatohepatitis in a Chinese population. World J. Gastroenterol..

[24] Salvoza N.C., Klinzing D.C., Gopez-Cervantes J., Baclig M.O. (2016). Association of Circulating Serum miR-34a and miR-122 with Dyslipidemia among Patients with Non-Alcoholic Fatty Liver Disease. PLoS ONE.

[25] Turchinovich A., Baranova A., Drapkina O., Tonevitsky A. (2018). Cell-free circulating nucleic acids as early biomarkers for NAFLD and NAFLD-associated disorders. Front. Physiol..

[26] Atic A.I., Thiele M., Munk A., Dalgaard L.T. (2023). Circulating miRNAs associated with nonalcoholic fatty liver disease. Am. J. Physiol. Cell Physiol..

[27] Popa M.L., Ichim C., Anderco P., Todor S.B., Pop-Lodromanean D. (2025). Micrornas in the diagnosis of digestive diseases: A comprehensive review. J. Clin. Med..

[28] Chatterjee B., Sarkar M., Bose S., Alam M.T., Chaudhary A.A., Dixit A.K., Tripathi P.P., Srivastava A.K. (2024). MicroRNAs: Key modulators of inflammation-associated diseases. Semin. Cell Dev. Biol..

[29] Yamada H., Suzuki K., Ichino N., Ando Y., Sawada A., Osakabe K., Sugimoto K., Ohashi K., Teradaira R., Inoue T. (2013). Associations between circulating microRNAs (miR-21, miR-34a, miR-122 and miR-451) and non-alcoholic fatty liver. Clin. Chim. Acta Int. J. Clin. Chem..

[30] Becker P.P., Rau M., Schmitt J., Malsch C., Hammer C., Bantel H., Müllhaupt B., Geier A. (2015). Performance of Serum microRNAs -122, -192 and -21 as Biomarkers in Patients with Non-Alcoholic Steatohepatitis. PLoS ONE.

[31] Pirola C.J., Fernández Gianotti T., Castaño G.O., Mallardi P., San Martino J., Mora Gonzalez Lopez Ledesma M., Flichman D., Mirshahi F., Sanyal A.J., Sookoian S. (2015). Circulating microRNA signature in non-alcoholic fatty liver disease: From serum non-coding RNAs to liver histology and disease pathogenesis. Gut.

[32] Huang X., Kong N., Zhang X., Cao Y., Langer R., Tao W. (2022). The landscape of mRNA nanomedicine. Nat. Med..

[33] Ito D., Kawaguchi Y., Inagaki Y., Ito K., Mihara Y., Kaneko J., Tanaka M., Fukayama M., Kokudo N., Hasegawa K. (2023). Assessment of liver function-related mRNA expression and fluorescence imaging in outflow-obstructed regions in rats. Surg. Today.

[34] Verschuren L., Mak A.L., van Koppen A., Özsezen S., Difrancesco S., Caspers M.P.M., Snabel J., van der Meer D., van Dijk A.-M., Rashu E.B. (2024). Development of a novel non-invasive biomarker panel for hepatic fibrosis in MASLD. Nat. Commun..

[35] Mattick J.S., Amaral P.P., Carninci P., Carpenter S., Chang H.Y., Chen L.-L., Chen R., Dean C., Dinger M.E., Fitzgerald K.A. (2023). Long non-coding RNAs: Definitions, functions, challenges and recommendations. Nat. Rev. Mol. Cell Biol..

[36] Coppin L., Leclerc J., Vincent A., Porchet N., Pigny P. (2018). Messenger RNA life-cycle in cancer cells: Emerging role of conventional and non-conventional RNA-binding proteins?. Int. J. Mol. Sci..

[37] Zeng Q., Liu C.-H., Wu D., Jiang W., Zhang N., Tang H. (2023). LncRNA and circRNA in patients with non-alcoholic fatty liver disease: A systematic review. Biomolecules.

[38] Zhou W., Qiu K. (2022). The correlation between lncRNA NEAT1 and serum hepcidin in the peripheral blood of non-alcoholic fatty liver disease patients. Am. J. Transl. Res..

[39] Shen X., Guo H., Xu J., Wang J. (2019). Inhibition of lncRNA HULC improves hepatic fibrosis and hepatocyte apoptosis by inhibiting the MAPK signaling pathway in rats with nonalcoholic fatty liver disease. J. Cell. Physiol..

[40] Hu M.-J., Long M., Dai R.-J. (2022). Acetylation of H3K27 activated lncRNA NEAT1 and promoted hepatic lipid accumulation in non-alcoholic fatty liver disease via regulating miR-212-5p/GRIA3. Mol. Cell. Biochem..

[41] Park J.G., Kim G., Jang S.Y., Lee Y.R., Lee E., Lee H.W., Han M.-H., Chun J.M., Han Y.S., Yoon J.S. (2020). Plasma Long Noncoding RNA LeXis is a Potential Diagnostic Marker for Non-Alcoholic Steatohepatitis. Life.

[42] Friedrich M., Aigner A. (2022). Therapeutic siRNA: State-of-the-art and future perspectives. BioDrugs.

[43] Lyu Y., Yang X., Yang L., Dai J., Qin H., Zhou Y., Huang Y., Wang Y., Wu D., Shuai Q. (2024). Lipid nanoparti-cle-mediated hepatocyte delivery of siRNA and silibinin in metabolic dysfunction-associated steatotic liver disease. J. Control. Release.

[44] Alberts B., Johnson A., Lewis J., Raff M., Roberts K., Walter P. (2002). From RNA to Protein. Molecular Biology of the Cell.

[45] Huang P., Tu B., Liao H.-J., Huang F.-Z., Li Z.-Z., Zhu K.-Y., Dai F., Liu H.-Z., Zhang T.-Y., Sun C.-Z. (2021). Elevation of plasma tRNA fragments as a promising biomarker for liver fibrosis in nonalcoholic fatty liver disease. Sci. Rep..

[46] Kristensen L.S., Andersen M.S., Stagsted L.V.W., Ebbesen K.K., Hansen T.B., Kjems J. (2019). The biogenesis, biology and characterization of circular RNAs. Nat. Rev. Genet..

[47] Bose R., Ain R. (2018). Regulation of Transcription by Circular RNAs. Adv. Exp. Med. Biol..

[48] Zhao Q., Liu J., Deng H., Ma R., Liao J.-Y., Liang H., Hu J., Li J., Guo Z., Cai J. (2020). Targeting Mitochondria-Located circRNA SCAR Alleviates NASH via Reducing mROS Output. Cell.

[49] Huang X., Yuan T., Tschannen M., Sun Z., Jacob H., Du M., Liang M., Dittmar R.L., Liu Y., Liang M. (2013). Characterization of human plasma-derived exosomal RNAs by deep sequencing. BMC Genom..

[50] Spanos M., Gokulnath P., Chatterjee E., Li G., Varrias D., Das S. (2023). Expanding the horizon of EV-RNAs: LncRNAs in EVs as biomarkers for disease pathways. Extracell. Vesicle.

[51] Li G., Manning A.C., Bagi A., Yang X., Gokulnath P., Spanos M., Howard J., Chan P.P., Sweeney T., Kitchen R. (2022). Distinct Stress-Dependent Signatures of Cellular and Extracellular tRNA-Derived Small RNAs. Adv. Sci..

[52] Thompson D.M., Parker R. (2009). Stressing out over tRNA cleavage. Cell.

[53] Zeng Q., Liu C.-H., Ampuero J., Wu D., Jiang W., Zhou L., Li H., Bai L., Romero-Gómez M., Tang H. (2024). Circular RNAs in non-alcoholic fatty liver disease: Functions and clinical significance. RNA Biol..

[54] Yu W., Hurley J., Roberts D., Chakrabortty S.K., Enderle D., Noerholm M., Breakefield X.O., Skog J.K. (2021). Exosome-based liquid biopsies in cancer: Opportunities and challenges. Ann. Oncol..

[55] Valadi H., Ekström K., Bossios A., Sjöstrand M., Lee J.J., Lötvall J.O. (2007). Exosome-mediated transfer of mRNAs and microRNAs is a novel mechanism of genetic exchange between cells. Nat. Cell Biol..

[56] Hu W., Liu C., Bi Z.-Y., Zhou Q., Zhang H., Li L.-L., Zhang J., Zhu W., Song Y.-Y.-Y., Zhang F. (2020). Comprehensive landscape of extracellular vesicle-derived RNAs in cancer initiation, progression, metastasis and cancer immunology. Mol. Cancer.

[57] Brandes F., Meidert A.S., Kirchner B., Yu M., Gebhardt S., Steinlein O.K., Dolch M.E., Rantner B., Tsilimparis N., Schelling G. (2024). Identification of microRNA biomarkers simultaneously expressed in circulating extracellular vesicles and atherosclerotic plaques. Front. Cardiovasc. Med..

[58] Arroyo J.D., Chevillet J.R., Kroh E.M., Ruf I.K., Pritchard C.C., Gibson D.F., Mitchell P.S., Bennett C.F., Pogosova-Agadjanyan E.L., Stirewalt D.L. (2011). Argonaute2 complexes carry a population of circulating microRNAs independent of vesicles in human plasma. Proc. Natl. Acad. Sci. USA.

[59] Xu L., Yang B., Ai J. (2013). MicroRNA transport: A new way in cell communication. J. Cell. Physiol..

[60] LaPlante E.L., Stürchler A., Fullem R., Chen D., Starner A.C., Esquivel E., Alsop E., Jackson A.R., Ghiran I., Pereira G. (2023). exRNA-eCLIP intersection analysis reveals a map of extracellular RNA binding proteins and associated RNAs across major human biofluids and carriers. Cell Genom..

[61] Vickers K.C., Palmisano B.T., Shoucri B.M., Shamburek R.D., Remaley A.T. (2011). MicroRNAs are transported in plasma and delivered to recipient cells by high-density lipoproteins. Nat. Cell Biol..

[62] Tabet F., Vickers K.C., Cuesta Torres L.F., Wiese C.B., Shoucri B.M., Lambert G., Catherinet C., Prado-Lourenco L., Levin M.G., Thacker S. (2014). HDL-transferred microRNA-223 regulates ICAM-1 expression in endothelial cells. Nat. Commun..

[63] Scicali R., Di Pino A., Pavanello C., Ossoli A., Strazzella A., Alberti A., Di Mauro S., Scamporrino A., Urbano F., Filippello A. (2019). Analysis of HDL-microRNA panel in heterozygous familial hypercholesterolemia subjects with LDL receptor null or defective mutation. Sci. Rep..

[64] Zhang H., Freitas D., Kim H.S., Fabijanic K., Li Z., Chen H., Mark M.T., Molina H., Martin A.B., Bojmar L. (2018). Identification of distinct nanoparticles and subsets of extracellular vesicles by asymmetric flow field-flow fractionation. Nat. Cell Biol..

[65] Anand S., Samuel M., Mathivanan S. (2021). Exomeres: A New Member of Extracellular Vesicles Family. Sub-Cell. Biochem..

[66] Clancy J.W., Boomgarden A.C., D’Souza-Schorey C. (2021). Profiling and promise of supermeres. Nat. Cell Biol..

[67] Zhang Q., Jeppesen D.K., Higginbotham J.N., Graves-Deal R., Trinh V.Q., Ramirez M.A., Sohn Y., Neininger A.C., Taneja N., McKinley E.T. (2021). Supermeres are functional extracellular nanoparticles replete with disease biomarkers and therapeutic targets. Nat. Cell Biol..

[68] Al Amir Dache Z., Otandault A., Tanos R., Pastor B., Meddeb R., Sanchez C., Arena G., Lasorsa L., Bennett A., Grange T. (2020). Blood contains circulating cell-free respiratory competent mitochondria. FASEB J..

[69] Stephens O.R., Grant D., Frimel M., Wanner N., Yin M., Willard B., Erzurum S.C., Asosingh K. (2020). Characterization and origins of cell-free mitochondria in healthy murine and human blood. Mitochondrion.

[70] Park J.-H., Hayakawa K. (2021). Extracellular Mitochondria Signals in CNS Disorders. Front. Cell Dev. Biol..

[71] Vikramdeo K.S., Anand S., Khan M.A., Khushman M., Heslin M.J., Singh S., Singh A.P., Dasgupta S. (2022). Detection of mitochondrial DNA mutations in circulating mitochondria-originated extracellular vesicles for potential diagnostic applications in pancreatic adenocarcinoma. Sci. Rep..

[72] Spanos M., Gokulnath P., Whittaker O.R., Azzam C., Chatterjee E., Singh A., Varrias D., Kladas M., Vulugundam G., Raptis D. (2024). Circulating Extracellular Mitochondria in Cardiometabolic Disease: Harnessing the Potential for Diagnosis, Prognosis, and Treatment. Physiologia.

[73] Chou S.H.Y., Lan J., Esposito E., Ning M., Balaj L., Ji X., Lo E.H., Hayakawa K. (2017). Extracellular Mitochondria in Cerebrospinal Fluid and Neurological Recovery After Subarachnoid Hemorrhage. Stroke.

[74] Sadik N., Cruz L., Gurtner A., Rodosthenous R.S., Dusoswa S.A., Ziegler O., Van Solinge T.S., Wei Z., Salvador-Garicano A.M., Gyorgy B. (2018). Extracellular RNAs: A New Awareness of Old Perspectives. Methods Mol. Biol..

[75] Zhou Z., Wu Q., Yan Z., Zheng H., Chen C.-J., Liu Y., Qi Z., Calandrelli R., Chen Z., Chien S. (2019). Extracellular RNA in a single droplet of human serum reflects physiologic and disease states. Proc. Natl. Acad. Sci. USA.

[76] Noninvasive Preeclampsia Prediction Using Plasma Cell–Free RNA Signatures—ClinicalKey. https://www.clinicalkey.com/#!/content/playContent/1-s2.0-S000293782300323X?returnurl=https:%2F%2Flinkinghub.elsevier.com%2Fretrieve%2Fpii%2FS000293782300323X%3Fshowall%3Dtrue&referrer=.

[77] Zhong P., Bai L., Hong M., Ouyang J., Wang R., Zhang X., Chen P. (2024). A Comprehensive Review on Circulating cfRNA in Plasma: Implications for Disease Diagnosis and Beyond. Diagnostics.

[78] Rozowsky J., Kitchen R.R., Park J.J., Galeev T.R., Diao J., Warrell J., Thistlethwaite W., Subramanian S.L., Milosavljevic A., Gerstein M. (2019). exceRpt: A Comprehensive Analytic Platform for Extracellular RNA Profiling. Cell Syst..

[79] Murillo O.D., Thistlethwaite W., Rozowsky J., Subramanian S.L., Lucero R., Shah N., Jackson A.R., Srinivasan S., Chung A., Laurent C.D. (2019). ExRNA atlas analysis reveals distinct extracellular RNA cargo types and their carriers present across human biofluids. Cell.

[80] Chatterjee E., Rodosthenous R.S., Kujala V., Gokulnath P., Spanos M., Lehmann H.I., de Oliveira G.P., Shi M., Miller-Fleming T.W., Li G. (2023). Circulating extracellular vesicles in human cardiorenal syndrome promote renal injury in a kidney-on-chip system. JCI Insight.

[81] Gokulnath P., Spanos M., Lehmann H.I., Sheng Q., Rodosthenous R., Chaffin M., Varrias D., Chatterjee E., Hutchins E., Li G. (2024). Distinct Plasma Extracellular Vesicle Transcriptomes in Acute Decompensated Heart Failure Subtypes: A Liquid Biopsy Approach. Circulation.

[82] Happel C., Ganguly A., Tagle D.A. (2020). Extracellular RNAs as potential biomarkers for cancer. J. Cancer Metastasis Treat..

[83] Tang R., Zhang Z., Xu J., Wang W., Meng Q., Liu Y., Du Q., Liang C., Hua J., Zhang B. (2024). Integration of single-nucleus and exosome RNA sequencing dissected inter-cellular communication and biomarkers in pancreatic ductal adenocarcinoma. Comput. Struct. Biotechnol. J..

[84] Luo T., Chen S.-Y., Qiu Z.-X., Miao Y.-R., Ding Y., Pan X.-Y., Li Y., Lei Q., Guo A.-Y. (2022). Transcriptomic Features in a Single Extracellular Vesicle via Single-Cell RNA Sequencing. Small Methods.

[85] Kojabad A.A., Farzanehpour M., Galeh H.E.G., Dorostkar R., Jafarpour A., Bolandian M., Nodooshan M.M. (2021). Droplet digital PCR of viral DNA/RNA, current progress, challenges, and future perspectives. J. Med. Virol..

[86] Wang C., Ding Q., Plant P., Basheer M., Yang C., Tawedrous E., Krizova A., Boulos C., Farag M., Cheng Y. (2019). Droplet digital PCR improves urinary exosomal miRNA detection compared to real-time PCR. Clin. Biochem..

[87] Campomenosi P., Gini E., Noonan D.M., Poli A., D’Antona P., Rotolo N., Dominioni L., Imperatori A. (2016). A comparison between quantitative PCR and droplet digital PCR technologies for circulating microRNA quantification in human lung cancer. BMC Biotechnol..

[88] Del Re M., Biasco E., Crucitta S., Derosa L., Rofi E., Orlandini C., Miccoli M., Galli L., Falcone A., Jenster G.W. (2017). The Detection of Androgen Receptor Splice Variant 7 in Plasma-derived Exosomal RNA Strongly Predicts Resistance to Hormonal Therapy in Metastatic Prostate Cancer Patients. Eur. Urol..

[89] Kuhlmann K., Cieselski M., Schumann J. (2021). Relative versus absolute RNA quantification: A comparative analysis based on the example of endothelial expression of vasoactive receptors. Biol. Proced. Online.

[90] Huang Z., Fang J., Zhou M., Gong Z., Xiang T. (2022). CRISPR-Cas13: A new technology for the rapid detection of pathogenic microorganisms. Front. Microbiol..

[91] Grölz D., Hauch S., Schlumpberger M., Guenther K., Voss T., Sprenger-Haussels M., Oelmüller U. (2018). Liquid biopsy preservation solutions for standardized pre-analytical workflows—Venous whole blood and plasma. Curr. Pathobiol. Rep..

[92] Asleh K., Dery V., Taylor C., Davey M., Djeungoue-Petga M.-A., Ouellette R.J. (2023). Extracellular vesicle-based liquid biopsy biomarkers and their application in precision immuno-oncology. Biomark. Res..

[93] De Sousa K.P., Rossi I., Abdullahi M., Ramirez M.I., Stratton D., Inal J.M. (2023). Isolation and characterization of extracellular vesicles and future directions in diagnosis and therapy. Wiley Interdiscip. Rev. Nanomed. Nanobiotechnol..

[94] Konoshenko M.Y., Lekchnov E.A., Vlassov A.V., Laktionov P.P. (2018). Isolation of extracellular vesicles: General methodologies and latest trends. BioMed Res. Int..

[95] Lone S.N., Nisar S., Masoodi T., Singh M., Rizwan A., Hashem S., El-Rifai W., Bedognetti D., Batra S.K., Haris M. (2022). Liquid biopsy: A step closer to transform diagnosis, prognosis and future of cancer treatments. Mol. Cancer.

[96] Zhu L., Sun H.-T., Wang S., Huang S.-L., Zheng Y., Wang C.-Q., Hu B.-Y., Qin W., Zou T.-T., Fu Y. (2020). Isolation and characterization of exosomes for cancer research. J. Hematol. Oncol..

[97] Hartjes T.A., Mytnyk S., Jenster G.W., van Steijn V., van Royen M.E. (2019). Extracellular vesicle quantification and characterization: Common methods and emerging approaches. Bioengineering.

[98] Masago K., Fujita S., Oya Y., Takahashi Y., Matsushita H., Sasaki E., Kuroda H. (2021). Comparison between fluorimetry (Qubit) and spectrophotometry (NanoDrop) in the quantification of DNA and RNA extracted from frozen and FFPE tissues from lung cancer patients: A real-world Use of genomic tests. Medicina.

[99] Conesa A., Madrigal P., Tarazona S., Gomez-Cabrero D., Cervera A., McPherson A., Szcześniak M.W., Gaffney D.J., Elo L.L., Zhang X. (2016). A survey of best practices for RNA-seq data analysis. Genome Biol..

[100] Brown J., Pirrung M., McCue L.A. (2017). FQC Dashboard: Integrates FastQC results into a web-based, interactive, and extensible FASTQ quality control tool. Bioinformatics.

[101] Babraham Bioinformatics—FastQC A Quality Control Tool for High Throughput Sequence Data. https://www.bioinformatics.babraham.ac.uk/projects/fastqc/.

[102] Raplee I.D., Evsikov A.V., Marín de Evsikova C. (2019). Aligning the aligners: Comparison of RNA sequencing data alignment and gene expression quantification tools for clinical breast cancer research. J. Pers. Med..

[103] Everaert C., Luypaert M., Maag J.L.V., Cheng Q.X., Dinger M.E., Hellemans J., Mestdagh P. (2017). Benchmarking of RNA-sequencing analysis workflows using whole-transcriptome RT-qPCR expression data. Sci. Rep..

[104] Patro R., Duggal G., Love M.I., Irizarry R.A., Kingsford C. (2017). Salmon: Fast and bias-aware quantification of transcript expression using dual-phase inference. Nat. Methods.

[105] Kolberg L., Raudvere U., Kuzmin I., Adler P., Vilo J., Peterson H. (2023). g:Profiler—Interoperable web service for functional enrichment analysis and gene identifier mapping (2023 update). Nucleic Acids Res..

[106] Brouard J.-S., Bissonnette N., Ng C., Piscuoglio S. (2022). Variant calling from RNA-seq data using the GATK joint genotyping workflow. Variant Calling: Methods and Protocols.

[107] Hochreuter M.Y., Dall M., Treebak J.T., Barrès R. (2022). MicroRNAs in non-alcoholic fatty liver disease: Progress and perspectives. Mol. Metab..

[108] Panella R., Petri A., Desai B.N., Fagoonee S., Cotton C.A., Nguyen P.K., Lundin E.M., Wagshal A., Wang D.-Z., Näär A.M. (2023). MicroRNA-22 is a key regulator of lipid and metabolic homeostasis. Int. J. Mol. Sci..

[109] Carpi S., Daniele S., de Almeida J.F.M., Gabbia D. (2024). Recent advances in miRNA-based therapy for MASLD/MASH and MASH-associated HCC. Int. J. Mol. Sci..

[110] Raptis D.D., Mantzoros C.S., Polyzos S.A. (2023). Fibroblast growth factor-21 as a potential therapeutic target of nonalcoholic fatty liver disease. Ther. Clin. Risk Manag..

[111] Gim J.-A., Bang S.M., Lee Y.-S., Lee Y., Yim S.Y., Jung Y.K., Kim H., Kim B.-H., Kim J.H., Seo Y.S. (2021). Evaluation of the severity of nonalcoholic fatty liver disease through analysis of serum exosomal miRNA expression. PLoS ONE.

[112] Cheung O., Puri P., Eicken C., Contos M.J., Mirshahi F., Maher J.W., Kellum J.M., Min H., Luketic V.A., Sanyal A.J. (2008). Nonalcoholic steatohepatitis is associated with altered hepatic MicroRNA expression. Hepatology.

[113] Povero D., Eguchi A., Li H., Johnson C.D., Papouchado B.G., Wree A., Messer K., Feldstein A.E. (2014). Circulating extracellular vesicles with specific proteome and liver micrornas are potential biomarkers for liver injury in experimental fatty liver disease. PLoS ONE.

[114] Kim T.H., Lee Y., Lee Y.-S., Gim J.-A., Ko E., Yim S.Y., Jung Y.K., Kang S., Kim M.Y., Kim H. (2021). Circulating miRNA is a useful diagnostic biomarker for nonalcoholic steatohepatitis in nonalcoholic fatty liver disease. Sci. Rep..

[115] Ando Y., Yamazaki M., Yamada H., Munetsuna E., Fujii R., Mizuno G., Ichino N., Osakabe K., Sugimoto K., Ishikawa H. (2019). Association of circulating miR-20a, miR-27a, and miR-126 with non-alcoholic fatty liver disease in general population. Sci. Rep..

[116] Akuta N., Kawamura Y., Suzuki F., Saitoh S., Arase Y., Kunimoto H., Sorin Y., Fujiyama S., Sezaki H., Hosaka T. (2016). Impact of circulating miR-122 for histological features and hepatocellular carcinoma of nonalcoholic fatty liver disease in Japan. Hepatol. Int..

[117] Tobaruela-Resola A.L., Riezu-Boj J.I., Milagro F.I., Mogna-Pelaez P., Herrero J.I., Elorz M., Benito-Boillos A., Tur J.A., Martínez J.A., Abete I. (2025). Circulating microRNA panels in subjects with metabolic dysfunction-associated steatotic liver disease after following a 2-year dietary intervention. J. Endocrinol. Investig..

[118] Liu J., Yang D., Wang B., Zeng Y., Li W. (2021). The value of miRNAs in the prognosis of obese patients receiving bariatric surgery. Am. J. Transl. Res..

[119] Li Y.-J., Baumert B.O., Stratakis N., Goodrich J.A., Wu H.-T., He J.-X., Zhao Y.-Q., Aung M.T., Wang H.-X., Eckel S.P. (2024). Circulating microRNA expression and nonalcoholic fatty liver disease in adolescents with severe obesity. World J. Gastroenterol..

[120] Aspichueta P., Zeisel M.B. (2023). miR-21p-5p coordinates biological pathways to promote MASLD progression. Liver Int..

[121] Tan Y., Ge G., Pan T., Wen D., Gan J. (2014). A pilot study of serum microRNAs panel as potential biomarkers for diagnosis of nonalcoholic fatty liver disease. PLoS ONE.

[122] Xiang J., Deng Y.-Y., Liu H.-X., Pu Y. (2022). LncRNA MALAT1 Promotes PPARα/CD36-Mediated Hepatic Lipogenesis in Nonalcoholic Fatty Liver Disease by Modulating miR-206/ARNT Axis. Front. Bioeng. Biotechnol..

[123] Leti F., Legendre C., Still C.D., Chu X., Petrick A., Gerhard G.S., DiStefano J.K. (2017). Altered expression of MALAT1 lncRNA in nonalcoholic steatohepatitis fibrosis regulates CXCL5 in hepatic stellate cells. Transl. Res..

[124] Tsai W.-C., Hsu S.-D., Hsu C.-S., Lai T.-C., Chen S.-J., Shen R., Huang Y., Chen H.-C., Lee C.-H., Tsai T.-F. (2012). MicroRNA-122 plays a critical role in liver homeostasis and hepatocarcinogenesis. J. Clin. Investig..

[125] Hsu S.-H., Wang B., Kota J., Yu J., Costinean S., Kutay H., Yu L., Bai S., La Perle K., Chivukula R.R. (2012). Essential metabolic, anti-inflammatory, and anti-tumorigenic functions of miR-122 in liver. J. Clin. Investig..

[126] Paluschinski M., Kordes C., Vucur M., Buettner V., Roderburg C., Xu H.C., Shinte P.V., Lang P.A., Luedde T., Castoldi M. (2023). Differential Modulation of miR-122 Transcription by TGFβ1/BMP6: Implications for Nonresolving Inflammation and Hepatocarcinogenesis. Cells.

[127] Colaianni F., Zelli V., Compagnoni C., Miscione M.S., Rossi M., Vecchiotti D., Di Padova M., Alesse E., Zazzeroni F., Tessitore A. (2024). Role of Circulating microRNAs in Liver Disease and HCC: Focus on miR-122. Genes.

[128] Wen J., Friedman J.R. (2012). miR-122 regulates hepatic lipid metabolism and tumor suppression. J. Clin. Investig..

[129] Xu Y., Zhu Y., Hu S., Pan X., Bawa F.C., Wang H.H., Wang D.Q.-H., Yin L., Zhang Y. (2021). Hepatocyte miR-34a is a key regulator in the development and progression of non-alcoholic fatty liver disease. Mol. Metab..

[130] Wang L., Sun M., Cao Y., Ma L., Shen Y., Velikanova A.A., Li X., Sun C., Zhao Y. (2020). miR-34a regulates lipid metabolism by targeting SIRT1 in non-alcoholic fatty liver disease with iron overload. Arch. Biochem. Biophys..

[131] Liu R.-H., Ning B., Ma X.-E., Gong W.-M., Jia T.-H. (2016). Regulatory roles of microRNA-21 in fibrosis through interaction with diverse pathways (Review). Mol. Med. Rep..

[132] Takeuchi-Yorimoto A., Yamaura Y., Kanki M., Ide T., Nakata A., Noto T., Matsumoto M. (2016). MicroRNA-21 is associated with fibrosis in a rat model of nonalcoholic steatohepatitis and serves as a plasma biomarker for fibrotic liver disease. Toxicol. Lett..

[133] Caviglia J.M., Yan J., Jang M.-K., Gwak G.-Y., Affo S., Yu L., Olinga P., Friedman R.A., Chen X., Schwabe R.F. (2018). MicroRNA-21 and Dicer are dispensable for hepatic stellate cell activation and the development of liver fibrosis. Hepatology.

[134] Markovic J., Sharma A.D., Balakrishnan A. (2020). MicroRNA-221: A Fine Tuner and Potential Biomarker of Chronic Liver Injury. Cells.

[135] Abdel-Al A., El-Ahwany E., Zoheiry M., Hassan M., Ouf A., Abu-Taleb H., Abdel Rahim A., El-Talkawy M.D., Zada S. (2018). miRNA-221 and miRNA-222 are promising biomarkers for progression of liver fibrosis in HCV Egyptian patients. Virus Res..

[136] Calvente C.J., Tameda M., Johnson C.D., Pilar H.d., Lin Y.C., Adronikou N., Jeu X.D.M.D., Llorente C., Boyer J., Feldstein A.E. (2019). Neutrophils contribute to spontaneous resolution of liver inflammation and fibrosis via microRNA-223. J. Clin. Investig..

[137] Gu J., Xu H., Chen Y., Li N., Hou X. (2022). MiR-223 as a Regulator and Therapeutic Target in Liver Diseases. Front. Immunol..

[138] Chen K., Lin T., Yao W., Chen X., Xiong X., Huang Z. (2023). Adipocytes-derived exosomal miR-122 promotes non-alcoholic fat liver disease progression via targeting Sirt1. Gastroenterol. Hepatol..

[139] Liu L., Xiao F., Sun J., Wang Q., Wang A., Zhang F., Li Z., Wang X., Fang Z., Qiao Y. (2023). Hepatocyte-derived extracellular vesicles miR-122-5p promotes hepatic ischemia reperfusion injury by regulating Kupffer cell polarization. Int. Immunopharmacol..

[140] Sun Y., Song Y., Liu C., Geng J. (2019). LncRNA NEAT1-MicroRNA-140 axis exacerbates nonalcoholic fatty liver through interrupting AMPK/SREBP-1 signaling. Biochem. Biophys. Res. Commun..

[141] Hussein M.A., Valinezhad K., Adel E., Munirathinam G. (2024). MALAT-1 Is a Key Regulator of Epithelial-Mesenchymal Transition in Cancer: A Potential Therapeutic Target for Metastasis. Cancers.

[142] Lu J., Guo J., Liu J., Mao X., Xu K. (2021). Long Non-coding RNA MALAT1: A Key Player in Liver Diseases. Front. Med..

[143] Sallam T., Jones M.C., Gilliland T., Zhang L., Wu X., Eskin A., Sandhu J., Casero D., Vallim T.Q.d.A., Hong C. (2016). Feedback modulation of cholesterol metabolism by the lipid-responsive non-coding RNA LeXis. Nature.

[144] Zhu J., Cheng M., Zhao X. (2020). A tRNA-derived fragment (tRF-3001b) aggravates the development of nonalcoholic fatty liver disease by inhibiting autophagy. Life Sci..

[145] Sohail A.M., Khawar M.B., Afzal A., Hassan A., Shahzaman S., Ali A. (2022). Multifaceted roles of extracellular RNAs in different diseases. Mil. Med. Res..

[146] Yamada H., Ohashi K., Suzuki K., Munetsuna E., Ando Y., Yamazaki M., Ishikawa H., Ichino N., Teradaira R., Hashimoto S. (2015). Longitudinal study of circulating miR-122 in a rat model of non-alcoholic fatty liver disease. Clin. Chim. Acta.

[147] Chai C., Rivkin M., Berkovits L., Simerzin A., Zorde-Khvalevsky E., Rosenberg N., Klein S., Yaish D., Durst R., Shpitzen S. (2017). Metabolic circuit involving free fatty acids, microRNA 122, and triglyceride synthesis in liver and muscle tissues. Gastroenterology.

[148] Chouik Y., Aubin A., Maynard-Muet M., Segrestin B., Milot L., Hervieu V., Zoulim F., Disse E., Levrero M., Caussy C. (2024). The grade of obesity affects the noninvasive diagnosis of advanced fibrosis in individuals with MASLD. Obesity.

[149] Angelini G., Panunzi S., Castagneto-Gissey L., Pellicanò F., Gaetano A.D., Pompili M., Riccardi L., Garcovich M., Raffaelli M., Ciccoritti L. (2022). Accurate liquid biopsy for the diagnosis of non-alcoholic steatohepatitis and liver fibrosis. Gut.

[150] Sanyal A.J., Williams S.A., Lavine J.E., Neuschwander-Tetri B.A., Alexander L., Ostroff R., Biegel H., Kowdley K.V., Chalasani N., Dasarathy S. (2023). Defining the serum proteomic signature of hepatic steatosis, inflammation, ballooning and fibrosis in non-alcoholic fatty liver disease. J. Hepatol..

[151] Chalasani N., Younossi Z., Lavine J.E., Charlton M., Cusi K., Rinella M., Harrison S.A., Brunt E.M., Sanyal A.J. (2018). The diagnosis and management of nonalcoholic fatty liver disease: Practice guidance from the American Association for the Study of Liver Diseases. Hepatology.

[152] Younossi Z.M., Loomba R., Anstee Q.M., Rinella M.E., Bugianesi E., Marchesini G., Neuschwander-Tetri B.A., Serfaty L., Negro F., Caldwell S.H. (2018). Diagnostic Modalities for Nonalcoholic Fatty Liver Disease, Nonalcoholic Steatohepatitis, and Associated Fibrosis. Hepatology.

[153] Castera L., Vilgrain V., Angulo P. (2013). Noninvasive evaluation of NAFLD. Nat. Rev. Gastroenterol. Hepatol..

[154] Hudson D., Afzaal T., Bualbanat H., AlRamdan R., Howarth N., Parthasarathy P., AlDarwish A., Stephenson E., Almahanna Y., Hussain M. (2024). Modernizing metabolic dysfunction-associated steatotic liver disease diagnostics: The progressive shift from liver biopsy to noninvasive techniques. Ther. Adv. Gastroenterol..

[155] Talwalkar J.A., Kurtz D.M., Schoenleber S.J., West C.P., Montori V.M. (2007). Ultrasound-based transient elastography for the detection of hepatic fibrosis: Systematic review and meta-analysis. Clin. Gastroenterol. Hepatol. Off. Clin. Pract. J. Am. Gastroenterol. Assoc..

[156] Yin M., Venkatesh S.K. (2018). Ultrasound or MR elastography of liver: Which one shall I use?. Abdom. Radiol..

[157] Shah A.G., Lydecker A., Murray K., Tetri B.N., Contos M.J., Sanyal A.J. (2009). Nash Clinical Research Network Comparison of noninvasive markers of fibrosis in patients with nonalcoholic fatty liver disease. Clin. Gastroenterol. Hepatol..

[158] Angulo P., Bugianesi E., Bjornsson E.S., Charatcharoenwitthaya P., Mills P.R., Barrera F., Haflidadottir S., Day C.P., George J. (2013). Simple noninvasive systems predict long-term outcomes of patients with nonalcoholic fatty liver disease. Gastroenterology.

[159] Dulai P.S., Sirlin C.B., Loomba R. (2016). MRI and MRE for non-invasive quantitative assessment of hepatic steatosis and fibrosis in NAFLD and NASH: Clinical trials to clinical practice. J. Hepatol..

[160] Chen J., Talwalkar J.A., Yin M., Glaser K.J., Sanderson S.O., Ehman R.L. (2011). Early detection of nonalcoholic steatohepatitis in patients with nonalcoholic fatty liver disease by using MR elastography. Radiology.

[161] Imajo K., Kessoku T., Honda Y., Tomeno W., Ogawa Y., Mawatari H., Fujita K., Yoneda M., Taguri M., Hyogo H. (2016). Magnetic resonance imaging more accurately classifies steatosis and fibrosis in patients with nonalcoholic fatty liver disease than transient elastography. Gastroenterology.

[162] Ferraioli G., Tinelli C., Dal Bello B., Zicchetti M., Filice G., Filice C. (2012). Liver Fibrosis Study Group Accuracy of real-time shear wave elastography for assessing liver fibrosis in chronic hepatitis C: A pilot study. Hepatology.

[163] Cui J., Heba E., Hernandez C., Haufe W., Hooker J., Andre M.P., Valasek M.A., Aryafar H., Sirlin C.B., Loomba R. (2016). Magnetic resonance elastography is superior to acoustic radiation force impulse for the Diagnosis of fibrosis in patients with biopsy-proven nonalcoholic fatty liver disease: A prospective study. Hepatology.

[164] Verma S., Jensen D., Hart J., Mohanty S.R. (2013). Predictive value of ALT levels for non-alcoholic steatohepatitis (NASH) and advanced fibrosis in non-alcoholic fatty liver disease (NAFLD). Liver Int..

[165] Anty R., Iannelli A., Patouraux S., Bonnafous S., Lavallard V.J., Senni-Buratti M., Amor I.B., Staccini-Myx A., Saint-Paul M.-C., Berthier F. (2010). A new composite model including metabolic syndrome, alanine aminotransferase and cytokeratin-18 for the diagnosis of non-alcoholic steatohepatitis in morbidly obese patients. Aliment. Pharmacol. Ther..

[166] Angulo P., Hui J.M., Marchesini G., Bugianesi E., George J., Farrell G.C., Enders F., Saksena S., Burt A.D., Bida J.P. (2007). The NAFLD fibrosis score: A noninvasive system that identifies liver fibrosis in patients with NAFLD. Hepatology.

[167] McPherson S., Hardy T., Dufour J.-F., Petta S., Romero-Gomez M., Allison M., Oliveira C.P., Francque S., Van Gaal L., Schattenberg J.M. (2017). Age as a confounding factor for the accurate non-invasive diagnosis of advanced NAFLD fibrosis. Am. J. Gastroenterol..

[168] Caviglia G.P., Casalone E., Rosso C., Aneli S., Allione A., Carli F., Grange C., Armandi A., Catalano C., Birolo G. (2025). Extracellular vesicles mirnome profiling reveals mirnas engagement in dysfunctional lipid metabolism, chronic inflammation and liver damage in subjects with metabolic dysfunction-associated steatotic liver disease. Aliment. Pharmacol. Ther..

[169] Beg M.S., Brenner A.J., Sachdev J., Borad M., Kang Y.-K., Stoudemire J., Smith S., Bader A.G., Kim S., Hong D.S. (2017). Phase I study of MRX34, a liposomal miR-34a mimic, administered twice weekly in patients with advanced solid tumors. Investig. New Drugs.

[170] Sato Y., Hatakeyama H., Sakurai Y., Hyodo M., Akita H., Harashima H. (2012). A pH-sensitive cationic lipid facilitates the delivery of liposomal siRNA and gene silencing activity in vitro and in vivo. J. Control. Release.

[171] Baldari S., Di Rocco G., Magenta A., Picozza M., Toietta G. (2019). Extracellular vesicles-encapsulated microRNA-125b produced in genetically modified mesenchymal stromal cells inhibits hepatocellular carcinoma cell proliferation. Cells.

[172] Anthiya S., Griveau A., Loussouarn C., Baril P., Garnett M., Issartel J.-P., Garcion E. (2018). MicroRNA-based drugs for brain tumors. Trends Cancer.

[173] Mishra S., Webster P., Davis M.E. (2004). PEGylation significantly affects cellular uptake and intracellular trafficking of non-viral gene delivery particles. Eur. J. Cell Biol..

[174] Yang J., Hendricks W., Liu G., McCaffery J.M., Kinzler K.W., Huso D.L., Vogelstein B., Zhou S. (2013). A nanoparticle formulation that selectively transfects metastatic tumors in mice. Proc. Natl. Acad. Sci. USA.

[175] Tobaruela-Resola A.L., Milagro F.I., Mogna-Pelaez P., Moreno-Aliaga M.J., Abete I., Zulet M.Á. (2025). The use of circulating miRNAs for the diagnosis, prognosis, and personalized treatment of MASLD. J. Physiol. Biochem..

[176] Zhu Y., Tan J.K., Wong S.K., Goon J.A. (2023). Therapeutic Effects of microRNAs on Nonalcoholic Fatty Liver Disease (NAFLD) and Nonalcoholic Steatohepatitis (NASH): A Systematic Review and Meta-Analysis. Int. J. Mol. Sci..

[177] Scholes A.N., Lewis J.A. (2020). Comparison of RNA isolation methods on RNA-Seq: Implications for differential expression and meta-analyses. BMC Genom..

[178] Evaluation of Serum Extracellular Vesicle Isolation Methods for Profiling miRNAs by Next-Generation Sequencing. https://pubmed-ncbi-nlm-nih-gov.elibrary.einsteinmed.edu/29887978/.

[179] Das S., Abdel-Mageed A.B., Adamidi C., Adelson P.D., Akat K.M., Alsop E., Ansel K.M., Arango J., Aronin N., Avsaroglu S.K. (2019). The Extracellular RNA Communication Consortium: Establishing Foundational Knowledge and Technologies for Extracellular RNA Research. Cell.

